# Transient *laminin beta* 1a Induction Defines the Wound Epidermis during Zebrafish Fin Regeneration

**DOI:** 10.1371/journal.pgen.1005437

**Published:** 2015-08-25

**Authors:** Chen-Hui Chen, Alexander F. Merriman, Jeremiah Savage, Jason Willer, Taylor Wahlig, Nicholas Katsanis, Viravuth P. Yin, Kenneth D. Poss

**Affiliations:** 1 Department of Cell Biology and Howard Hughes Medical Institute, Duke University School of Medicine, Durham, North Carolina, United States of America; 2 Center for Human Disease Modeling, Department of Cell Biology, Duke University, Durham, North Carolina, United States of America; 3 Davis Center for Regenerative Biology and Medicine, Mount Desert Island Biological Laboratory, Salisbury Cove, Maine, United States of America; University of Pennsylvania School of Medicine, UNITED STATES

## Abstract

The first critical stage in salamander or teleost appendage regeneration is creation of a specialized epidermis that instructs growth from underlying stump tissue. Here, we performed a forward genetic screen for mutations that impair this process in amputated zebrafish fins. Positional cloning and complementation assays identified a temperature-sensitive allele of the ECM component *laminin beta 1a* (*lamb1a*) that blocks fin regeneration. *lamb1a*, but not its paralog *lamb1b*, is sharply induced in a subset of epithelial cells after fin amputation, where it is required to establish and maintain a polarized basal epithelial cell layer. These events facilitate expression of the morphogenetic factors *shha* and *lef1*, basolateral positioning of phosphorylated Igf1r, patterning of new osteoblasts, and regeneration of bone. By contrast, *lamb1a* function is dispensable for juvenile body growth, homeostatic adult tissue maintenance, repair of split fins, or renewal of genetically ablated osteoblasts. *fgf20a* mutations or transgenic Fgf receptor inhibition disrupt *lamb1a* expression, linking a central growth factor to epithelial maturation during regeneration. Our findings reveal transient induction of *lamb1a* in epithelial cells as a key, growth factor-guided step in formation of a signaling-competent regeneration epidermis.

## Author Summary

Unlike mammals, adult teleost fish and urodele amphibians can fully regenerate lost appendages. Understanding what initiates regeneration in these vertebrates is of great interest to the scientific community. It has long been known that the epidermis that forms quickly over an amputated limb stump is critical for initiating regenerative programs. Yet, little of understood of the molecular and cellular mechanisms by which a simple adult epithelium transforms into this key signaling source. Here, we performed a large-scale, unbiased genetic screen for epithelial signaling deficiencies during the regeneration of amputated adult zebrafish fins, from which we identified several new mutants. One gene identified from this screen disrupts a specific component of the extracellular matrix material Laminin, Laminin beta 1a, a factor that we find to be dispensable in uninjured adult animals but required for all stages fin regeneration. Transient induction of this component by amputation polarizes the basal layer of the nascent epithelium, and, in turn, facilitates the synthesis of signaling factors, the positioning of ligand receptors, and the patterning of new bone cells. We also find that normal induction of Laminin beta 1a by injury relies on the function of Fibroblast growth factors, secreted polypeptide signals that are released early upon injury. Our results identify key early steps in the endogenous program for vertebrate appendage regeneration.

## Introduction

Mammals have a limited ability to regenerate complex structures like limbs, heart or central nervous system tissue. By contrast, teleost fish and urodele amphibians can regenerate major appendages, spinal cord, retina, brain, kidney, and the heart [1–7]. How and why tissue regeneration occurs in non-mammalian vertebrates has fascinated biologists for centuries and is relevant to regenerative medicine strategies.

Previous studies of appendage regeneration have identified three prominent phases: 1) wound healing; 2) blastema formation; and 3) regenerative outgrowth and patterning [8]. Upon amputation injury, the exposed stump tissue is rapidly covered by a sheet of epithelial cells, a process involving little or no cell proliferation [9, 10]. Classical studies in salamanders revealed that if this epithelium is removed, replaced with flank skin, or disrupted by insertion of the limb into the abdominal cavity, limb regeneration does not proceed [11–13]. The wound epithelium becomes multilayered and acquires a layer of cuboidal basal epithelial cells over the next hours to days, maturing into a structure commonly referred to as the wound or regeneration epidermis. Regeneration epidermises of salamander limbs or teleost fins are known to express markers of developmental signaling pathways, including many secreted factors [8, 14]. For instance, after initial epithelialization of an amputated zebrafish fin stump, epithelial and/or mesenchymal cells induce effectors of pathways mediated by Fgfs, Igfs, Wnts, Hhs, Activin-βA/TGFβ, Retinoic acid, Bmps, and Notch [15–27]. Interestingly, *fgf20a* and *igf2b* ligand genes are induced within hours of fin amputation in mesenchymal cells, and perturbation of Fgf signaling via a mutation in the *fgf20a* ligand gene, or of Igf signaling by receptor inhibition, disrupts formation of the regeneration epidermis and subsequent bone regeneration [20, 21]. These findings indicate an important role for early expression of growth factors in structural and functional maturation of epithelial tissue. However, mechanisms by which these pathways define the morphology and signaling activities of the regeneration epidermis have not been addressed.

Here, we used forward genetics to identify a critical role for *laminin beta 1a* (*lamb1a*), one of two paralogs encoding a Laminin beta 1 extracellular matrix component, in zebrafish fin regeneration. *lamb1a* is sharply induced upon fin amputation in the basal layer of the wound epithelium, where its function is required to establish polarity in basal epithelial cells, induce and maintain basal epithelial markers, localize receptors for signaling, and align regenerating osteoblasts. *lamb1a* induction is dependent on Fgf signaling, both immediately after amputation and throughout regeneration. Thus, Lamb1a is a critical node between growth factor signals and formation of the regeneration epidermis in an amputated vertebrate appendage.

## Results

### Forward genetic screen for epithelial signaling defects during fin regeneration

**Fig 1:**
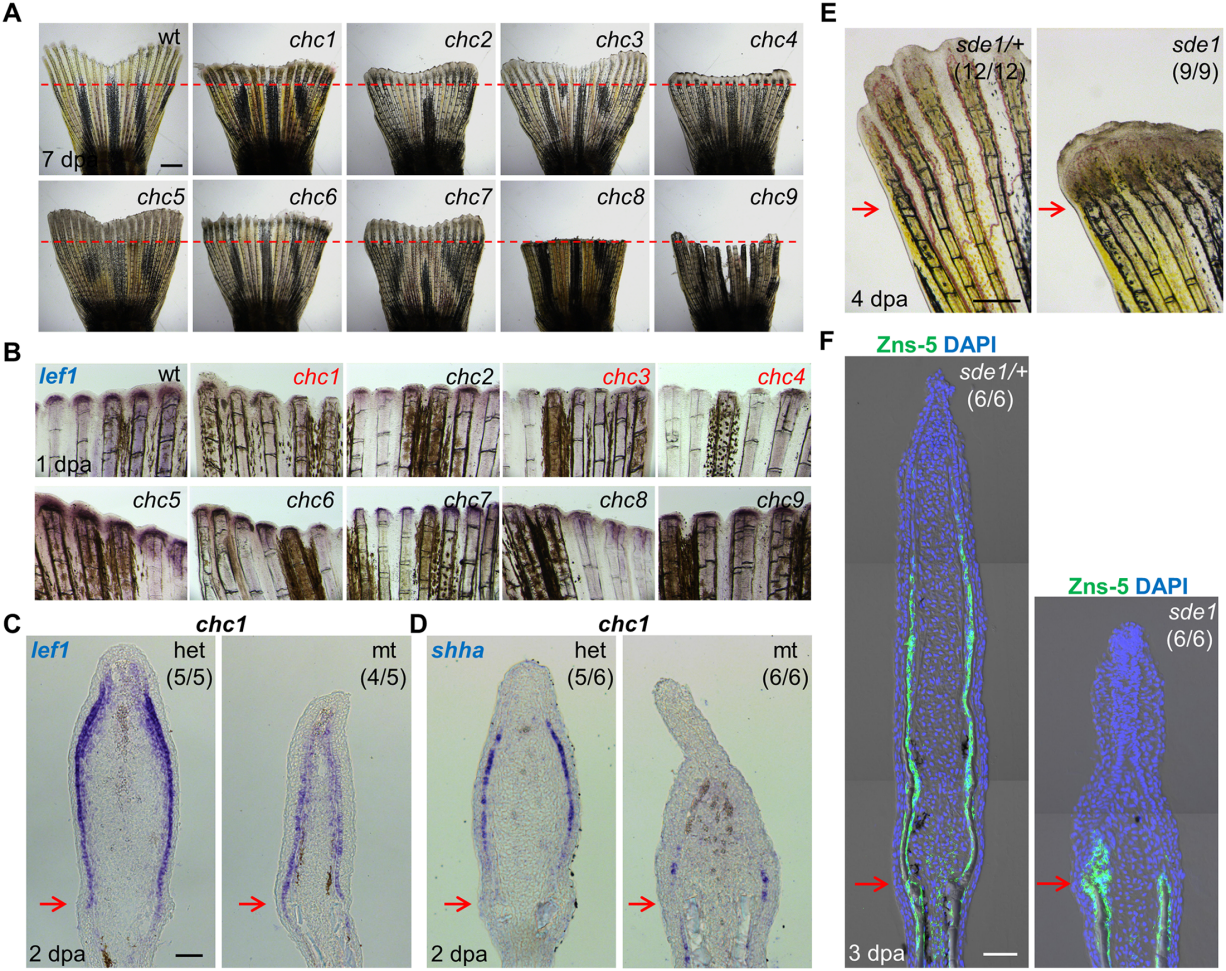
Forward genetic screen for signaling defects in the fin regeneration epidermis. (A) Whole-mount images of wild-type and (*chc1-9*) mutant regenerates at 7 days post amputation (dpa). Red dashed lines indicate plane of amputation. Scale bars, 1 mm. (B) Whole-mount RNA ISH of *lef1* expression in wild-type and mutant regenerates at 1 dpa. *chc1*, *chc3*, and *chc4* mutant families show reduced or undetectable *lef1* expression when compared to their respective heterozygous siblings. (C, D) Longitudinal sections of 2 dpa fin regenerates assessed by RNA ISH, showing reduced *lef1* and *shha* in *chc1* mutant regenerates. (E) Whole-mount images of *sde1* (formerly, *chc1*) fin regenerates at 4 dpa. Scale bars, 0.5 mm. (F) Longitudinal sections of 3 dpa fin regenerates show impaired patterning of osteoblasts in *sde1* (*chc1*) mutants, assessed by Zns-5 antibody staining (green). DAPI, blue. Scale bars, 50 μm (unless otherwise indicated). Red arrows indicate plane of amputation.

Previous genetic screens for mutants in zebrafish fin regeneration involved parthenogenesis of F1 generation females [1, 28]. This approach saves considerable time and animal facility space, as progeny with homozygous ENU-induced mutations can be screened in the F2 generation. Yet, it also limits animal survival and access to chromosomal regions far from centromeres [29]. For this study, we conducted a three-generation screen, in which we raised 423 F3 families from 108 F2 generation crosses to adulthood at a temperature of 25°C. To identify temperature-sensitive mutants that can be used for toggling gene function, we shifted these adults to 33°C after amputating ~50% of the caudal fins, and then assessed regeneration 7 days later. After several rounds of outcrossing to identify stable phenotypes and dilute unlinked ENU mutations from the genetic background, we found 9 families (*chc1-9*) with temperature-sensitive defects in fin regeneration inherited as a single recessive determinant (Fig 1A).

To identify a subset of mutants that disrupt formation of a functional regeneration epidermis, we examined *lef1* expression at 1 dpa by in situ RNA hybridization (ISH; Fig 1B). *lef1* is a downstream effector and transcriptional target of Wnt signaling that is induced in wound epithelial cells adjacent to the amputation plane as early as 12 hours post-amputation [30]. Three mutant lines, *chc1*, *chc3*, and *chc4*, consistently displayed reduced *lef1* expression at 1 dpa (Fig 1B), from which we initially pursued *chc1*. *chc1* regenerates also had markedly reduced expression of the Hedgehog ligand *shha*, which, like *lef1*, is induced in basal epithelial cells (Fig 1C and 1D). *shha* has been implicated in blastemal cell proliferation and alignment of osteoblasts to areas of prospective bone during zebrafish fin regeneration [15, 31].

**Fig 2:**
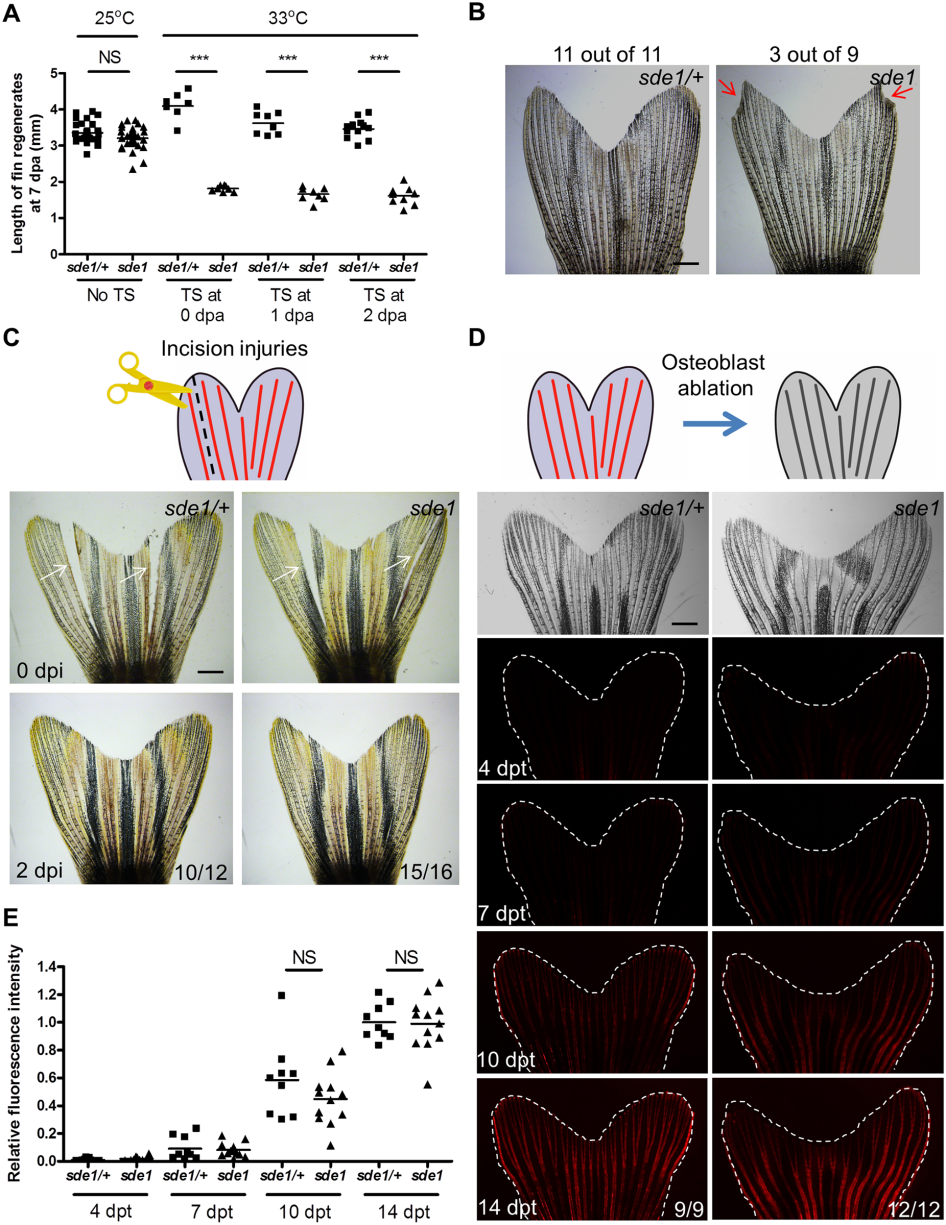
*sde1* requirements for tissue regeneration depend on injury context. (A) Measurement of *sde1* fin regenerates at 7 dpa. After amputation, animals were shifted from the permissive temperature (25°C) to the restrictive temperature (33°C) at 0, 1 or 2 dpa. TS, temperature shift. (n = 15, 16, and 21; Student’s *t* -test, ***P < 0.001; NS, non-significant). (B) Adult *sde1/+* and *sde1* animals were incubated at 33°C for two months (n = 11 and 9). Red arrows point to a damaged fin edge in *sde1*. The most severe example of damage in *sde1* animals is displayed here (3 of 9 showed damage in the experiment). (C) (Top) Cartoon depicting the model of incision injuries. (Bottom) Whole-mount images were acquired at 0 and 2 days post incision injury (dpi). Images from the same animal are shown before (top) and after repair (bottom). White arrows indicate sites of injury (n = 12 and 16). (D) (Top) Cartoon depicting the model of osteoblast ablation. (Bottom) Fluorescence intensity, indicating recovery of genetically labeled osteoblasts after ablation, was recorded at 4, 7, 10, and 14 days post Mtz treatment (dpt) and quantified using ImageJ software. White dashed lines indicate fin boundaries. Images from the same animal are shown throughout recovery. (E) Quantification of relative fluorescence intensity from individual animals after osteoblasts ablation (n = 9 and 12; mean ± SEM; Student’s *t*-test; NS, non-significant). Scale bars, 1 mm.

*chc1* mutants, renamed *signaling deficient epidermis 1* (*sde1*) mutants, regenerated amputated fins normally at 25°C, indicating a strictly temperature-sensitive effect. Inspection of *sde1* fin regenerates showed reduced lengths and no detectable bone at 4 dpa (Fig 1E). Osteoblasts typically begin to align adjacent to the basal epithelial layer by 2 dpa, where they deposit bone minerals that comprise ray hemisegments [1]. Immunofluorescence analysis of 3 dpa *sde1* tissue sections revealed the accumulation of osteoblasts in a mass adjacent to the epithelium, or limited presence at all, as opposed to an even distribution to lateral regions (Fig 1F). Analysis of 7 dpa *sde1* regenerates indicated reduced but clearly detectable osteoblast-lined bone, with osteoblasts accumulated in small masses at the distal regions of bone (S1A Fig). To determine whether *sde1* regenerates eventually reach full length, we assessed fin ray lengths at 14, 21, and 28 dpa. We observed visibly obvious and statistically significant regenerative defects at each of these time points (S1B and S1C Fig). Thus, *sde1* mutations have a long-lasting impact on regeneration. To determine temporal requirements for *sde1* during fin regeneration, we shifted the animals from 25°C to 33°C at 0, 1, or 2 dpa, and assessed fin lengths at 7 dpa. Each of these procedures resulted in significant defects in *sde1* regenerates (Fig 2A), indicative of continued requirements throughout various stages of regeneration. Thus, *sde1* is one of a subset of mutations that inhibits induction of morphogenetic signals in the regeneration epidermis. The *sde1* gene product is critical for osteoblast patterning, bone formation, and progression of regeneration.

### Injury context-specific requirements for *sde1*

Previous studies of fin regeneration mutants revealed distinct phenotypes when animals are placed at the restrictive temperature for long time periods. *sly1* and *hsp60* mutants survive poorly beyond 14 days at 33°C, likely reflecting roles in fundamental cell survival or organismal physiology [32, 33]. *fgf20a* and *mps1* mutants, as well as transgenic animals enabling prolonged expression of a dominant-negative Fgf receptor, each survive well at restrictive conditions but display progressive loss of 17–36% of distal fin tissue over a course of 60 days [34]. *sde1* mutants showed no adverse effects at 33°C over a 60-day period, displaying higher survival than any other new mutants identified in this study (100%, n = 36; S2 Fig). A minority of *sde1* mutants lost small amounts of distal fin tissue (3/9, Fig 2B). These experiments indicate minimal requirements for *sde1* in basic cell, tissue, or organismal function, or in homeostatic maintenance of fin structures.

To examine requirements in other forms of regeneration, we performed two additional injury models. First, we made a precise incision within interray tissue that spanned most of the proximodistal length of the caudal fin. This injury, which severed interray mesenchyme and epithelial cells but not bone, is typically healed within 2 days and most likely reflects simple wound-healing [21]. *sde1* mutants and heterozygous mutant siblings each rapidly healed these incisions (Fig 2C). Second, we introduced a transgene for visualizing and genetically ablating osteoblasts (*osx*:*mCherry-NTR*) into the *sde1* background. Adult *osx*:*mCherry-NTR* zebrafish quickly repopulate fin rays that have been depleted of virtually all osteoblasts after treatment with the pro-drug metronidazole [35]. Following osteoblast depletion in *sde1* mutants and heterozygous clutchmates, we quantified the recovery of *osx*-driven fluorescence intensity over 14 days (Fig 2D). We detected no defects in the ability of *sde1* mutants to regenerate osteoblasts in these experiments (Fig 2E). Thus, *sde1* mutations potently affect regeneration of amputated fins, but they have little or no effects on the ability of adult zebrafish to maintain fin tissue or regenerate complex fin injuries that do not require a regeneration epidermis.

### *sde1* encodes a *laminin beta1* paralog

**Fig 3:**
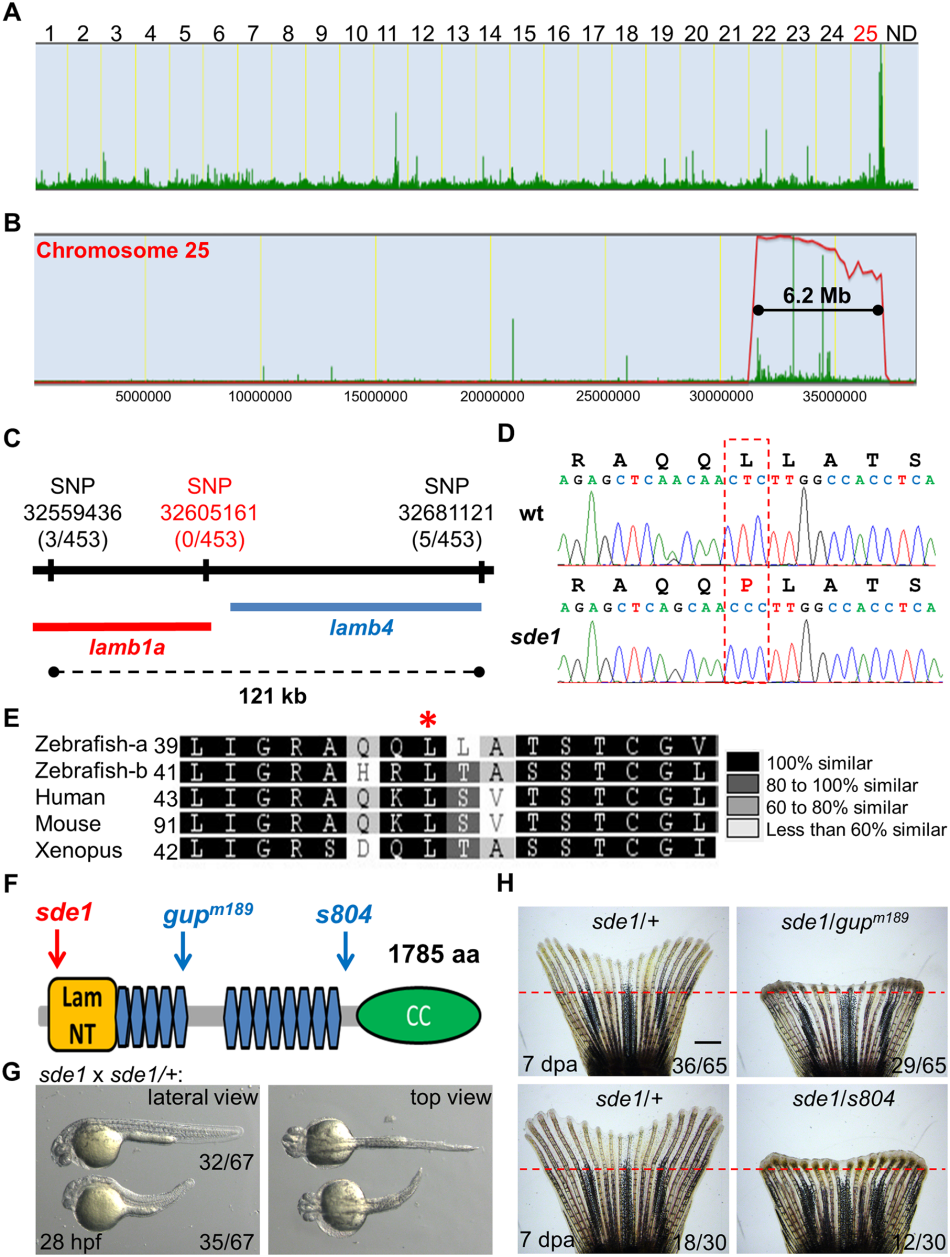
*sde1* encodes a *laminin beta1* paralog. (A) Analysis of genomic homozygosity in *sde1* mutants. (B) Log likelihood analysis using SNPtrack. (C) High-resolution mapping using linked SNP markers. Three and five recombinants were found for SNP32559436 and SNP32681121, respectively, on each side of a 121 kb region. After genotyping 453 adult animals, no recombinants were identified for a novel SNP at position 32605161. (D) Sanger sequencing readouts from wild-type and *sde1* cDNA. SNP32605161 is within the coding region of the gene *laminin beta 1a* (*lamb1a*), causing a leucine to proline change. (E) Amino acid alignment across distant species. Red star marks the location of the leucine. Differential gray scale indicates level of conservation across listed species. (F) Cartoon depicting major structural domains in Lamb1a. Blue and red arrows indicate the locations of the *sde1* mutation, along with two previously identified alleles *gup* ^*m189*^ and *s804*. Lam NT, Laminin N-terminal domain; Blue hexagons, Laminin-type epidermal growth factor-like domain; CC, uncharacterized coiled-coil domain. (G) *sde1* embryos incubated at 31°C have shortened trunks. Representative embryos from an *sde1* x *sde1/+* cross. Embryos were transferred to 31°C at 3 hours post-fertilization (hpf). Images were acquired at 28 hpf. Approximately 48% of embryos (32 out of 67) showed phenotypes representative of the *gup* ^*m189*^ mutation after the temperature shift, consistent with expected Mendelian ratio. The phenotype and the ratio were consistent across three independent crosses. (H) Complementation tests showing both *gup* ^*m189*^ and *s804* alleles fail to complement the 7 dpa regeneration defects of the *sde1* mutation in adult animals, yielding expected ratios (~50%; n = 65 and 30). Red dashed lines indicate plane of amputation. Scale bars, 1 mm.

To identify the gene that is disrupted in *sde1* mutants, we performed whole-exome sequencing of clutchmate DNA from an *sde1* x *sde1/+* cross (see Materials and Methods) [36]. Sequencing data were analyzed using the web-based mapping tool SNPtrack, which facilitates linkage analysis based on single nucleotide polymorphisms (SNPs) and regions of homozygosity [37]. Primary analysis from SNPtrack revealed a single 6.2 Mb peak on chromosome 25 containing *sde1* (Fig 3A and 3B). We genotyped 453 adults from several *sde1* × *sde1/+* mapping crosses for polymorphic SNPs in this region. From this linkage analysis, we identified 3 closely linked SNPs that flanked a 121 kb region containing two genes: *laminin beta 1a* (*lamb1a*) and *laminin beta 4b* (*lamb4b*) (Fig 3C). By filtering against a SNP database established in the Poss lab (see Materials and Methods) and cDNA sequencing, we identified one novel non-synonymous mutation (T to C) in the coding region of the gene *lamb1a* causing a lysine to proline change at position 46 (Fig 3D). No coding mutations were identified in *lamb4b*. Zebrafish have two unlinked paralogs encoding *laminin beta 1*, *lamb1a* and *lamb1b*, emerging from a partial genome duplication event estimated at 350 million years ago [38]. Sequence analysis revealed that lysine 46 is conserved between these two zebrafish paralogs, and also among *laminin beta 1* genes in other vertebrate species like human and *Xenopus laevis* (Fig 3E). Additionally, the candidate *sde1* mutation is located in a highly conserved Laminin N-terminal (Lam NT) domain (Fig 3F), which has been demonstrated to mediate interactions with other Laminin members [39, 40]. Thus, genetic mapping associates *sde1* with a mutation in a conserved residue of the *laminin beta 1* paralog, *lamb1a*.

Two independent loss-of-function mutations in *lamb1a* have been isolated in zebrafish, *gup* ^*m189*^ and *s804* (Fig 3F) [41, 42]. We compared phenotypes of *sde1* embryos raised at 31°C to those described for the *gup* ^*m189*^ embryos, and found trunk shortening reminiscent of the *gup* ^*m189*^ phenotype in ~50% of 28 hpf embryos from mapping crosses (Fig 3G). Furthermore, to test whether *sde1* complements known *lamb1a* mutations, we crossed homozygous *sde1* mutants with either *gup* ^*m189*^ or *s804* heterozygous mutant zebrafish. Each of these crosses gave rise to ~50% of progeny with temperature-sensitive defects in adult fin regeneration (Fig 3H). Thus, based on high-resolution genetic mapping, expected embryonic phenotypes, and two independent complementation tests, we conclude that *sde1* encodes a conditional allele of *lamb1a* (*lamb1a* ^*pd110*^), most likely acting as a hypomorph at the restrictive temperature.

### *lamb1a*, but not *lamb1b*, is an induced component of the regeneration epidermis

**Fig 4:**
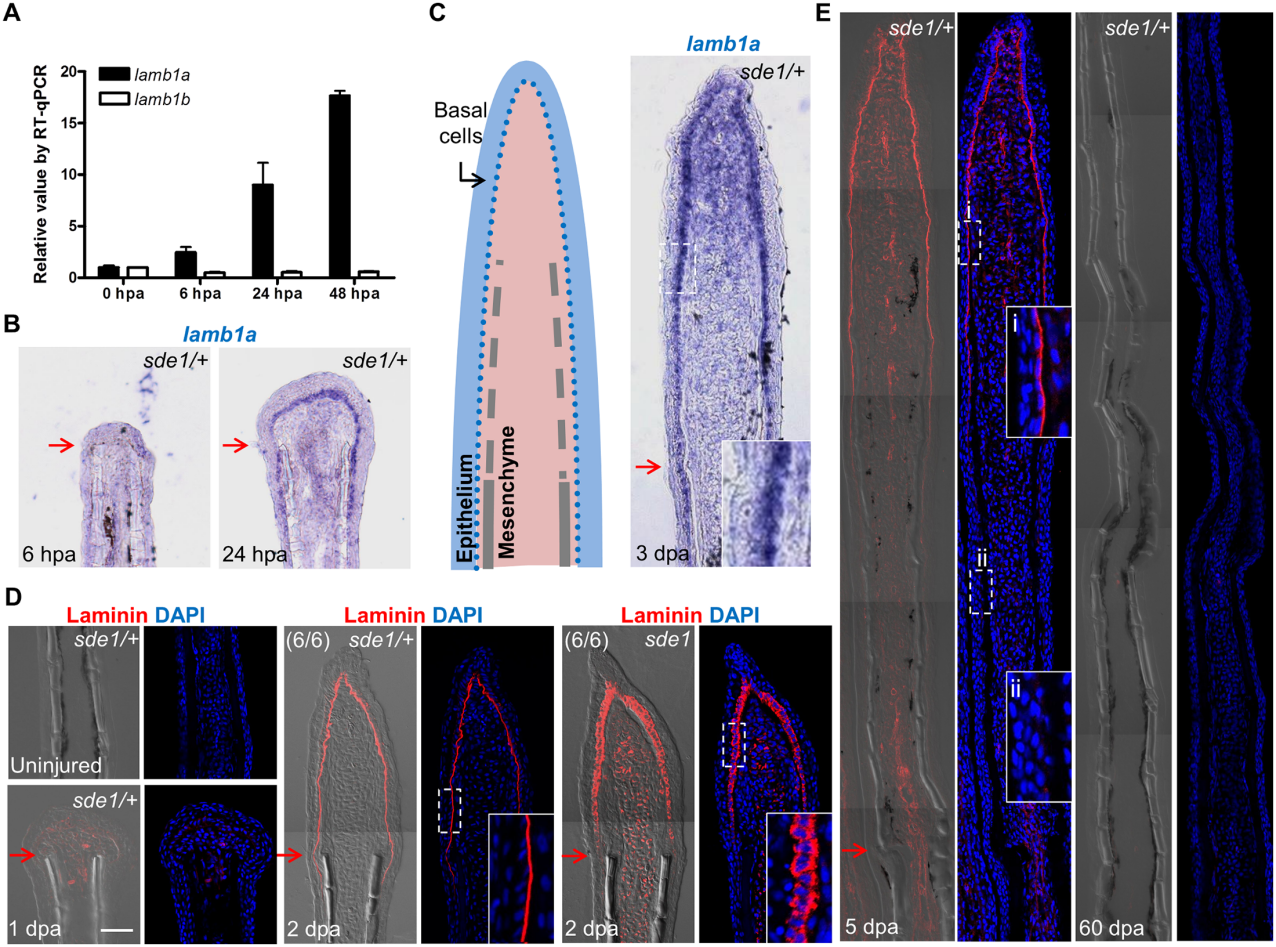
*lamb1a*, not *lamb1b*, is induced during regeneration. (A) RT-qPCR analysis indicates that *lamb1a*, but not *lamb1b*, is induced during regeneration. qPCR results were normalized to *rpl13a* and to the basal expression of *lamb1a*/*lamb1b* at 0 hours post-amputation (hpa). (n = 3; mean ± SEM). (B) Section ISH indicating that *lamba1* becomes visually detectable in the basal epithelial layer between 6 and 24 hpa. (C) Left: cartoon depicting basic cellular makeup of the fin regenerate. Right: *lamb1a* is expressed in basal epithelial cells and some mesenchymal cells at 3 dpa. (D) Antibody staining for Laminin expression in regenerating fins. Laminin protein is restricted to the basal side of the basal epithelial cells layer by 2 dpa in wild-type or *sde1/+* regenerates, but mislocalized to all regions of basal cells in *sde1* mutants. (E) Laminin expression at 5 dpa and 60 dpa, indicating that Laminin presence is transient during regenerative outgrowth. i: distal, newly regenerated tissue; ii: proximal regenerated tissue. Laminin, red; DAPI, blue. Scale bars, 50 μm. White dashed boxes indicate areas of enlarged view. Red arrows indicate plane of amputation.

Using specific qPCR probes targeting *lamb1a* and *lamb1b* sequences, we found that *lamb1a* expression, but not *lamb1b*, was induced during regeneration (Fig 4A). ISH experiments failed to detect *lamb1a* RNA in uninjured or 6 hpa fins, but visualized *lamb1a* by 1 dpa mainly in basal epithelial cells, and less prominently in mesenchymal cells (Fig 4B). During regenerative outgrowth, *lamb1a* expression was maintained in regenerating tissue in a primary basal epithelial cell domain and a secondary mesenchymal domain (Fig 4C). Using an antibody raised against mouse basement membrane Laminin, we found analogous expression domains for Laminin at the protein level. Laminin presence was undetectable in uninjured fins, and evident by 1 dpa (Fig 4D). By 2 dpa, Laminin was primarily localized to the basal side of the basal epithelial cell layer (Fig 4D), ostensibly part of the extracellular basement membrane. Laminin presence gradually waned proximal to the amputation plane from 2 to 5 dpa, and was undetectable in 60 dpa regenerates (Fig 4E). Thus, *lamb1a* is regulated differently from its *lamb1b* paralog upon fin amputation, an injury that induces *lamb1a* transiently in epithelial tissues and maintains its expression during key stages of regeneration.

### *lamb1a* is required for juvenile fin morphology but not body size

**Fig 5:**
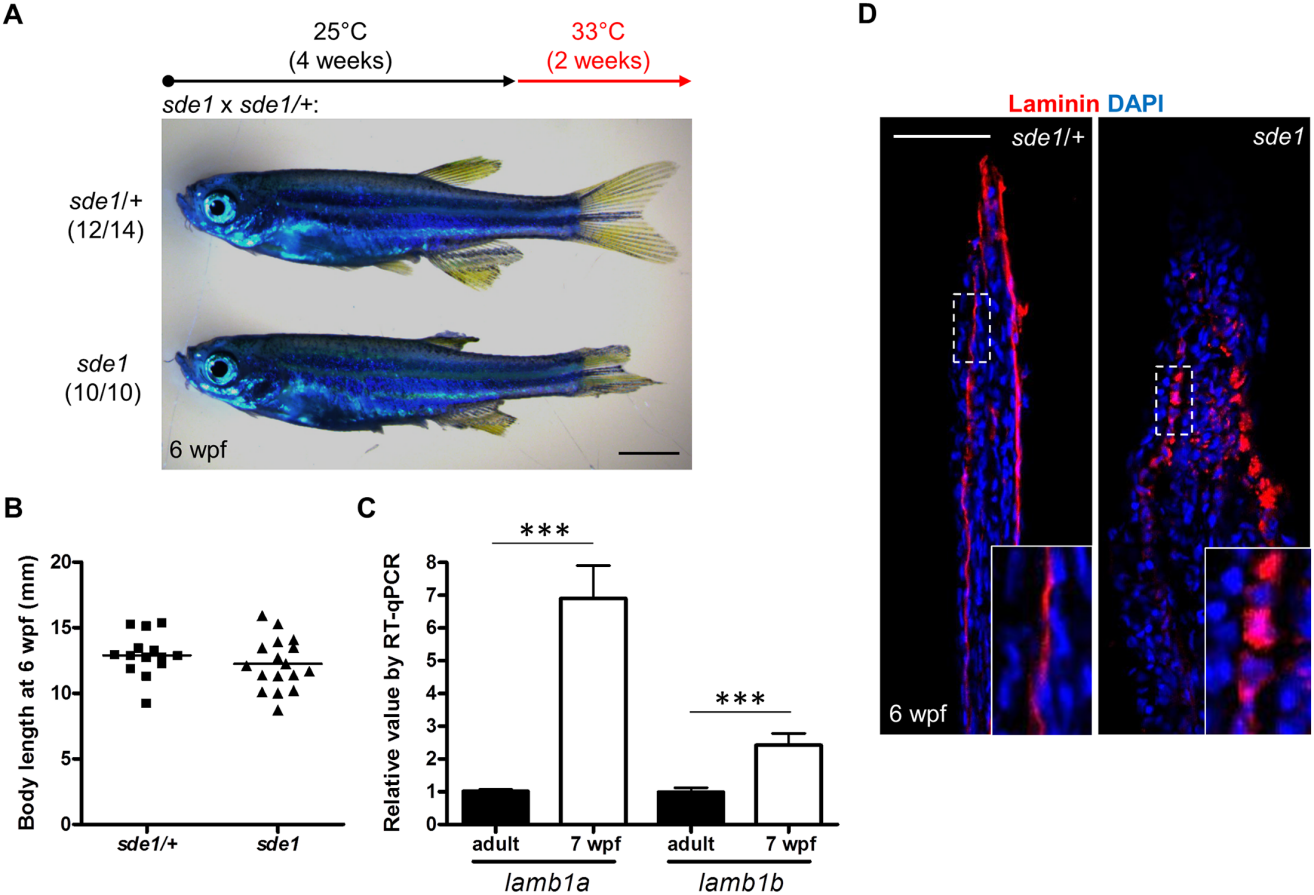
*lamb1a* is required for juvenile fin growth but not body growth. **(A)** Juvenile *sde1* animals, after incubation from 4 to 6 weeks post-fertilization (wpf) at 33°C, acquire degraded fins. Scale bars, 2 mm. (B) *sde1* mutations have minimal impact on juvenile outgrowth. Body length was measured from the tip of the snout to the base of caudal fin. (C) RT-qPCR analysis indicates that both *lamb1a* and *lamb1b* are induced in fin tissue during juvenile outgrowth. qPCR results were normalized to *rpl13a* and to the basal expression of *lamb1a/lamb1b* in adult uninjured fins (n = 4; mean ± SEM). (D) Antibody staining for Laminin expression in juvenile fins. Laminin protein in longitudinal sections of fins is localized to the basal side of the epitheilum in *sde1/+* animals, but becomes mislocalized in *sde1* mutants.

To examine *lamb1a* functions during juvenile growth, we shifted *sde1* animals from 25°C to 33°C at 4 weeks post fertilization (wpf), after zebrafish reach their juvenile stage. After 2 weeks at 33°C, all 6 wpf *sde1* animals (10/10) had noticeably degraded fins, whereas majority of *sde1/+* siblings displayed no noticeable fin phenotypes (12/14). Interestingly, this temperature shift did not grossly affect the body length of *sde1* juvenile animals (Fig 5B). Using specific qPCR probes targeting *lamb1a* and *lamb1b* sequences, we found that both *lamb1a* and *lamb1b* mRNA levels were higher in juvenile fins than in adult uninjured fins (Fig 5C). These results indicate that *lamb1a* is required for juvenile fin growth and/or tissue maintenance, but is dispensable for organismal growth at the juvenile stage.

### *lamb1a* induction is required to induce and maintain polarity and signaling in basal cells of the regeneration epidermis

**Fig 6:**
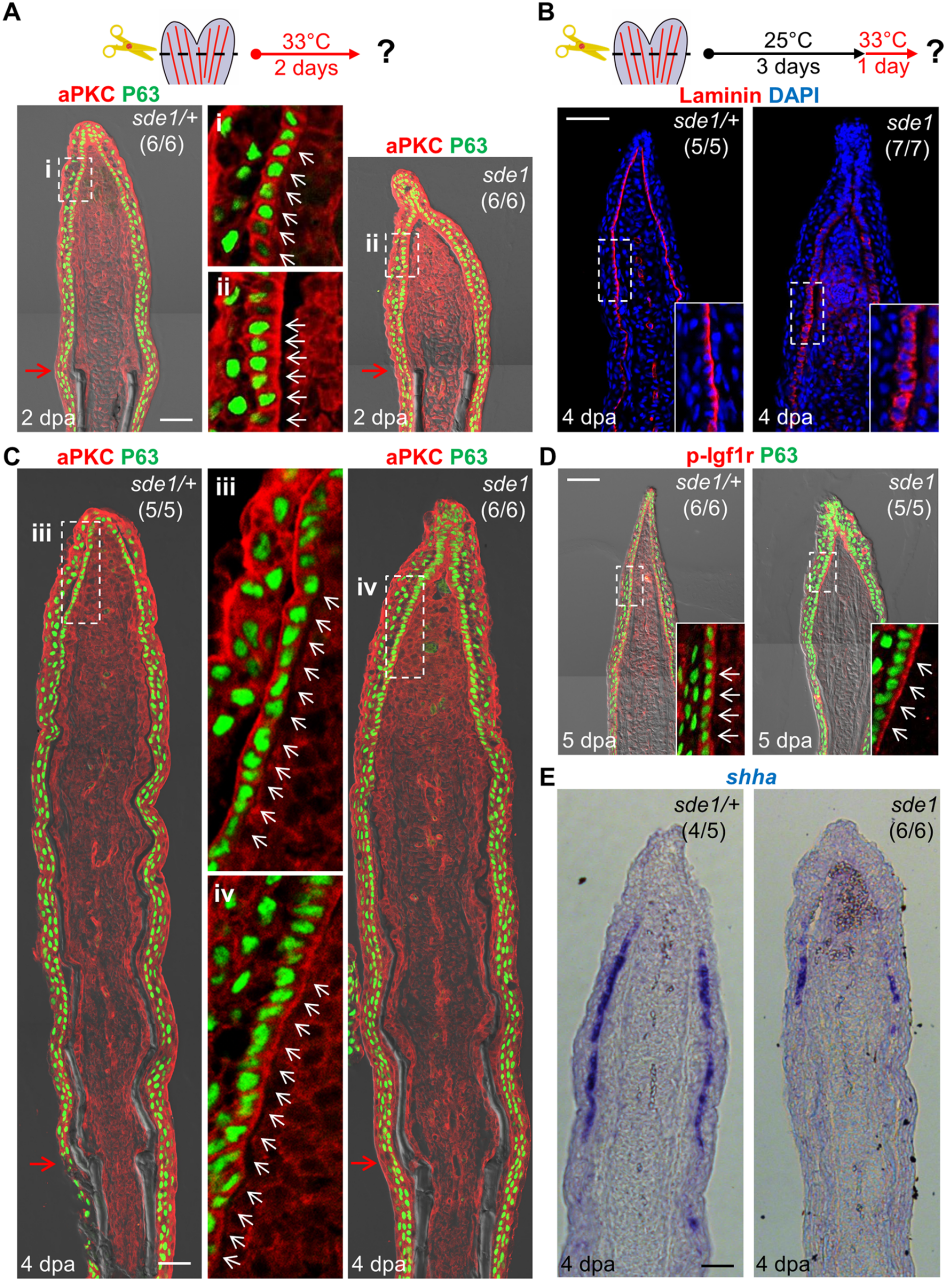
*lamb1a* induction defines cell polarity and signaling competence in basal cells of the regeneration epidermis. (A) Antibody co-staining for aPKC (red) and P63 (green; an epithelial maker for all basal and some suprabasal epithelial cells) in longitudinal sections of *sde1/+* and *sde1* fin regenerates at 2 dpa. (B) Antibody staining for Laminin in fin regenerates at 4 dpa after a temperature shift from 25°C to 33°C at 3 dpa, indicating induced mislocalization. Laminin, red; DAPI, blue. White dashed boxes indicate areas of enlarged view. (C) Antibody co-staining for aPKC (red) and P63 (green) in longitudinal sections of *sde1/+* and *sde1* fin regenerates at 4 dpa after a temperature shift from 25°C to 33°C at 3 dpa, indicating loss of basal cell polarity. iii: distal regenerated tissue (*sde1/+*); iv: distal regenerated tissue (*sde1*). White arrows indicate basal cell nuclei. (D) Antibody co-staining for phosphorylated Igf1r (red) and P63 (green) in longitudinal sections of 5 dpa *sde1/+* and *sde1* fin regenerates, after a temperature shift from 25°C to 33°C at 4 dpa. The basal localization of p-Igf1r is enriched in basal epithelial cells in *sde1* mutants. (E) *shha* RNA expression is reduced in *sde1* fin regenerates at 4 dpa after a temperature shift from 25°C to 33°C at 3 dpa. Scale bars, 50 μm. White dashed boxes indicate areas of enlarged view. Red arrows indicate plane of amputation.

Laminin is widely studied as a component of the basement membrane; thus, its induction in the basal epithelial layer strongly suggested a role in creating this structure. We examined Laminin presence in 2 dpa regenerates of *sde1* mutants at 33°C, and found it ectopically localized to all basal epithelial cell regions including the apical and lateral portions (Fig 4D), indicate of intracellular residence. This result supports the idea that Lamb1a interaction with other Laminin members through the N-terminal domain may be important for secretion of Laminin complexes [39, 40]. Laminin was also aberrantly localized in *sde1* juvenile fins maintained at 33°C for 14 days (Fig 5D). To examine the polarity of the basal epithelial layer at 2 dpa, we used an antibody raised against atypical Protein kinase C (aPKC), a well-characterized apical marker that is essential to maintain epithelial polarity in many systems, including nematodes, flies, and mammalian cells [43]. Whereas aPKC was localized to apical and lateral regions of basal epithelial cells by 2 dpa in wild-type fins, *sde1* regenerates accumulated aPKC expression in basal regions of this cell layer (Fig 6A). This finding is consistent with in vitro [44, 45] and in vivo [46] functional studies indicating Laminin as a polarizing cue for epithelial cells.

To determine whether Lamb1a function actively maintains epithelial cell polarity during regenerative outgrowth, we shifted *sde1* animals from 25°C to 33°C at 3 dpa, after the polarity of basal cells was established (Fig 6A). After one day at 33°C, Laminin and aPKC protein became mislocalized in basal epithelial cells (Fig 6B and 6C), indicating that Lamb1a normally maintains this polarity during outgrowth. To examine possible functional consequences of lost basal epithelial polarity, we examined the localization of a receptor of Insulin-like growth factor (Igf) signaling in basal cells of the regeneration epidermis. Igf1r is autophosphorylated in basal epithelial cells during fin regeneration, presumably after engagement by Igf2b and possibly other ligands, and its activity is required for fin regeneration [21]. Whereas phosphorylated Igf1r is located basolaterally in control fin regenerates, one day at 33°C demonstrably enriched the basal expression domain in *sde1* regenerates. This rapid change suggests that localization of phosphorylated Igf1r is actively maintained by components of the cell polarity machinery. Additionally, we found that *shha* expression visualized by ISH was consistently reduced in basal epithelial cells after shifting to 33°C for one day at 3 or 4 dpa (Fig 6E and S3 Fig). The function of Lamb1a in maintaining cell polarity and *shha* expression is unlikely to be a consequence of a general slowing of regeneration, as 8 hours of 33°C treatment at 3 dpa was sufficient to alter aPKC localization and reduce *shha* expression (S4 Fig). Additionally, a one-day temperature shift did not grossly decrease indicators of cell proliferation in mesenchymal cells (S5 Fig), suggesting that cell proliferation in one or more subpopulations of blastemal cells is not directly affected. Together, these experiments reveal that *lamb1a* induction has a central role in formation of a polarized regeneration epidermis after fin amputation. They also suggest that this epithelial polarization is critical for localization of signaling receptors, expression of key morphogenetic signals, osteoblast patterning, and bone regeneration.

### Fgf signaling influences *lamb1a* expression during regeneration

**Fig 7:**
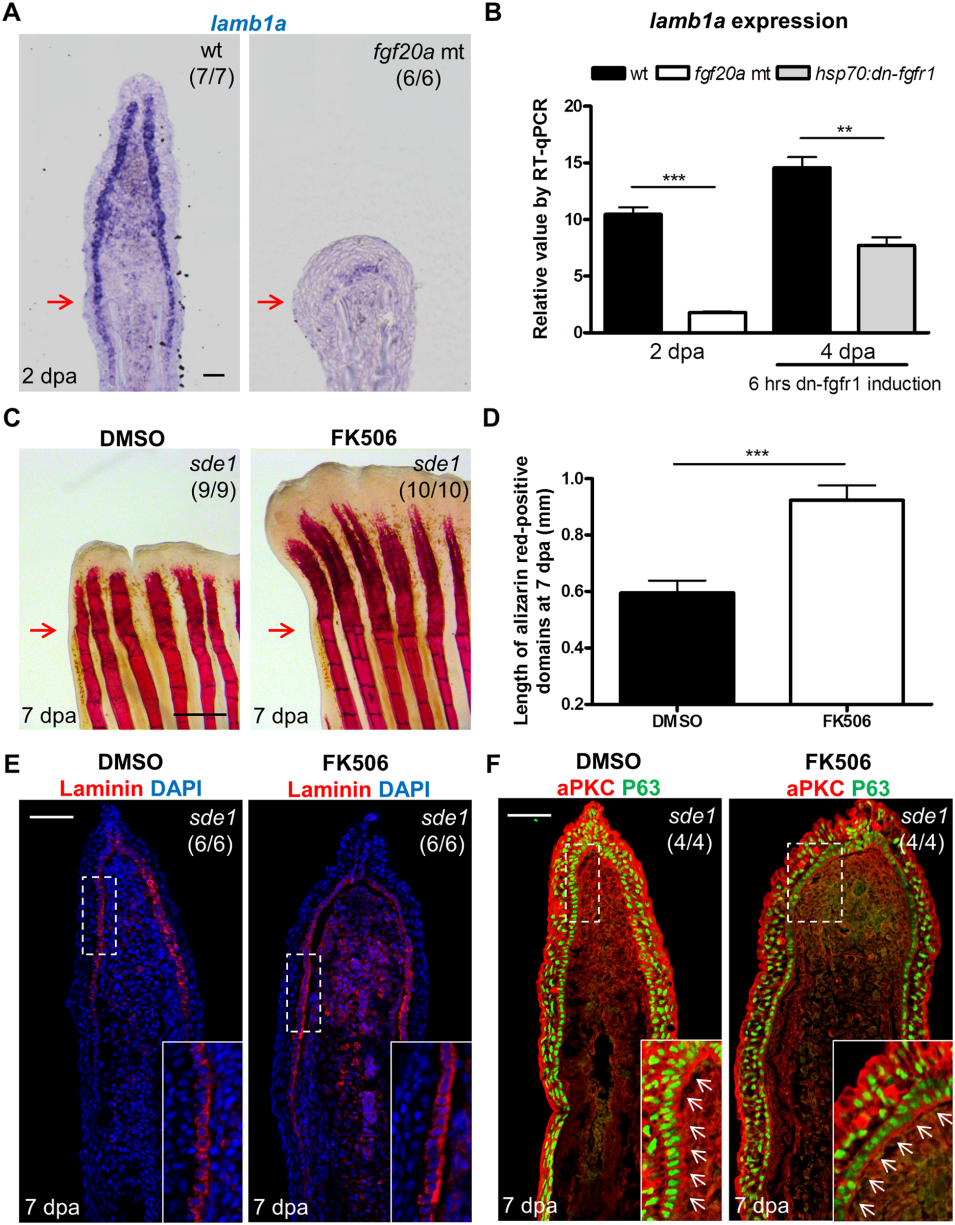
Association of *lamb1a* expression and function with key regeneration effector pathways. (A) Longitudinal sections of 2 dpa fin regenerates stained for *lamb1a* by ISH, indicating sparse expression in *fgf20a* mutants (*dob*). Scale bars, 50 μm. (B) RT-qPCR analysis indicating depleted levels of *lamb1a* RNA in *fgf20a* mutants (left). When Fgf signaling is blocked by induced expression of a dominant–negative Fgf receptor for just 6 hours at 4 dpa, *lamb1a* levels drop by nearly 50%. qPCR results were normalized to *rpl13a* and to the basal expression of *lamb1a* at 0 hpa. (n = 4; mean ± SEM; Student’s *t* -test, ***P < 0.001, **P < 0.01). (C) Whole-mount images of fin regenerates stained by alizarin red staining for calcium deposition, after treatment of *sde1* animals with DMSO or FK506. Scale bars, 0.5 mm. (D) Measurement of the length of alizarin red-positive domains at 7 dpa (n = 9 and 10; mean ± SEM; Student’s *t* -test, ***P < 0.001). (E) Antibody staining for Laminin protein in vehicle- or FK506-treated *sde1* fin regenerates at 7 dpa. Laminin, red; DAPI, blue. (F) Antibody co-staining for aPKC (red) and P63 (green) expression in vehicle or FK506-treated *sde1* fin regenerates at 7 dpa. FK506 treatment partially rescued bone regeneration in *sde1* mutants, with no detectable impact on Laminin localization or basal epithelial cell polarity. Scale bars, 50 μm. White dashed boxes indicate areas of enlarged view. White arrows indicate basal cell nuclei. Red arrows indicate plane of amputation.

Like *lamb1a*, the Fgf ligand gene *fgf20a* is induced during zebrafish fin regeneration. Moreover, *fgf20a* mutants are also defective in *lef1* induction and maturation of the regeneration epidermis [20]. Fgf signaling has been implicated in control of Laminin production in the context of embryoid bodies [47]. Because of these links, we investigated possible expression associations between *lamb1a* and *fgf20a*. We found that the induction of *fgf20a* was not affected in *sde1* regenerates (S6A Fig). By contrast, *lamb1a* induction was severely disrupted in *fgf20a* mutants at 2 dpa (Fig 7A and 7B), at which point Laminin protein was detectable at very low levels along the epithelial-mesenchymal boundary (S6B Fig). To determine whether Fgf signaling actively sustains *lamb1a* expression during regeneration, we employed a transgenic line *Tg(hsp70*:*dnfgfr1-EGFP)* ^*pd1*^ that drives a dominant negative Fgfr1 cassette from a heat-shock-inducible promoter [48]. A single heat-shock treatment at 4 dpa to transiently attenuate Fgf signaling during regeneration was sufficient to reduce *lamb1a* expression by 48% within 6 hours (Fig 7B), suggesting direct control of *lamb1a* at the transcriptional level by Fgf signaling. By contrast, 24 hours treatment from 3 to 4 dpa with either the Igf receptor antagonist NVP-AEW541 or the Igf signaling agonist NBI-31772 did not significant alter *lamb1a* expression (S6C Fig). Similarly, the induction of *lamb1a* was not affected in *sde1* regenerates at 2 dpa at the restrictive temperature, as assayed by qPCR (S6D Fig). These results implicate *fgf20a* upstream of *lamb1a* in activation of morphogenesis of the regeneration epidermis.

To test whether bone growth programs potentially downstream of epithelial *lamb1a* function could rescue regeneration, we treated *sde1* animals with the Calcineurin inhibitor FK506 during fin regeneration. A recent study reported that increases in Calcineurin activity reduce ray growth as regeneration progresses, a model supported by the finding that extended FK506 treatment causes gross lengthening of regenerated fin rays [27]. Interestingly, we found that FK506 treatment for 7 days increased the length of *sde1* regenerates (from 39% of untreated *sde1/+* regenerates at 7 dpa to 54%; S7A, S7B and S7C Fig), partially rescuing the length of regenerated bone (Fig 7C and 7D). By contrast, treatment of Smoothened Agonist (SAG) to activate the Hedgehog signaling pathway did not increase the lengths of *sde1* regenerates (S7D and S7E Fig). Notably, FK506 treatment had little or no impact on mislocalized Laminin and aPKC in *sde1* basal epithelial cells (Fig 7E and 7F). We were also unable to detect increased expression of *shha* in FK506-treated regenerates (S8 Fig). While partial rescue of length and bone formation occurred, FK506-treated *sde1* regenerates were clearly dysmorphic compared to clutchmate control regenerates (S7A Fig). Although it is possible that FK506 treatment acts independently of *lamb1a*-mediated functions, our findings indicate that restoration of some mesenchymal compartment osteogenesis may occur in the presence of epithelial defects.

## Discussion

Here, we carried out a forward genetic screen for zebrafish mutants defective in creation of a signaling-competent regeneration epidermis. The *sde1* product is not required to repopulate genetically ablated osteoblasts or heal incision wounds, and appears largely dispensable in uninjured adult animals. However, after fin amputation, *sde1* lesions disrupt epidermal maturation and signaling, and impair osteoblast patterning, and they remain inhibitory throughout the process of tissue replacement. Genetic analyses, including gene product assessment and complementation with known mutants, define *sde1* as a temperature-sensitive allele of *lamb1a*, an ECM component that is transiently induced by injury in a subset of epithelial cells. Thus, Lamb1a is a key component of the regeneration epidermis with context-specific roles in appendage regeneration.

**Fig 8:**
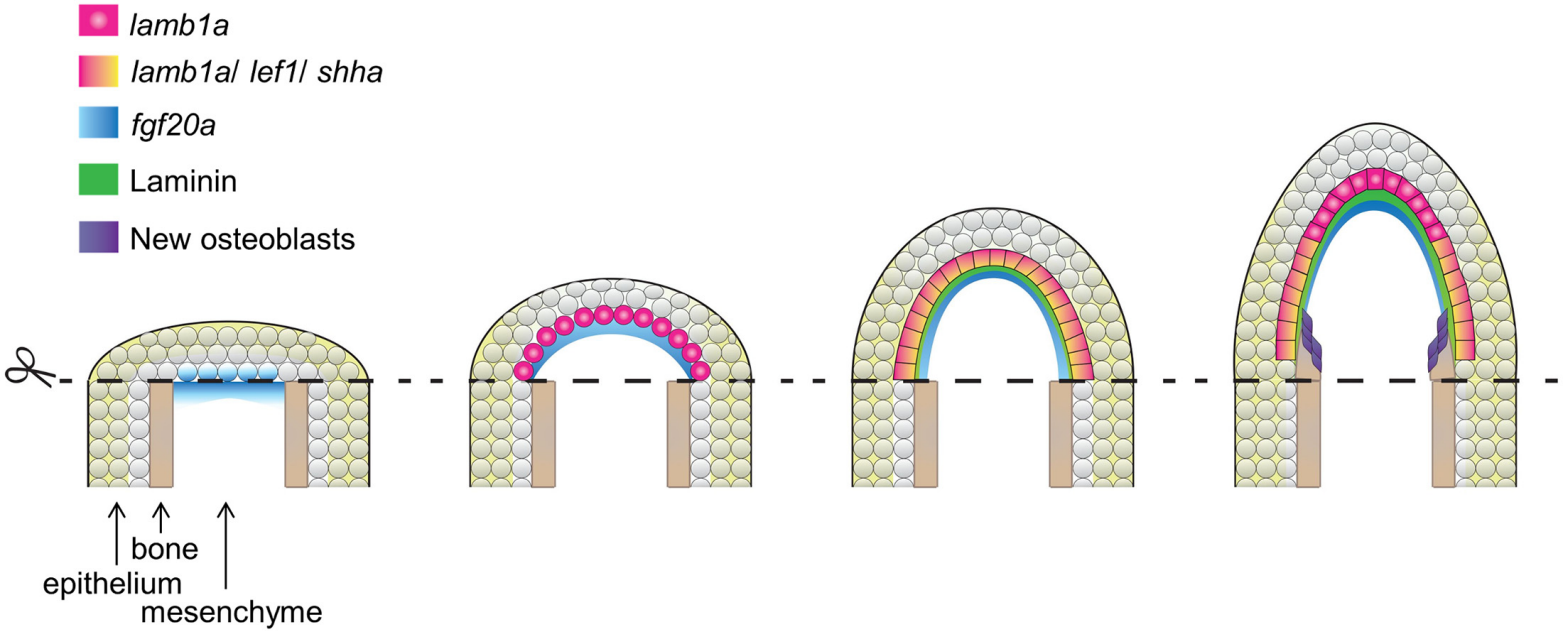
Model for morphogenesis of the regeneration epidermis. After initial wound closure, *fgf20a* is induced at the epithelial-mesenchymal boundary by 6 hours post-amputation. *fgf20a* signaling then contributes to induction of expression of the *lamb1a* paralog in the adjacent epithelial cell layer to establish a basement membrane and to polarize the basal cell layer. This polarization is essential for positioning signaling receptors and induction of morphogenetic factors that guide osteoblast patterning and bone formation.

Our findings, combined with those of past studies, suggest a regulatory model for construction and maintenance of the regeneration epidermis (Fig 8). After an amputation injury, the wound closes within the next several hours by epithelial cell migration, ostensibly controlled by tension changes within the epithelial sheet. *fgf20a* is induced at the epithelial-mesenchymal boundary by 6 hours post-amputation. One direct or indirect function of *fgf20a* signaling is to induce expression of the *lamb1a* paralog in the adjacent epithelial cell layer, which establishes a basement membrane and polarizes the basal cell layer. This polarization is essential for positioning signaling receptors, such as phosphorylated Igf1r, and induction of factors involved in Hh signaling and Wnt/β-catenin signaling (via indirect influences [17, 49]) that guide osteoblast patterning and bone formation. During regenerative outgrowth, Fgf signaling retains *lamb1a* expression in the basal cell layer, maintaining polarization in basal epithelial cells and the competence to signal to osteoblasts. Our results with FK506 indicate that Lamb1a function is predominantly epithelial, as activation of the mesenchymal component is sufficient to promote bone regeneration to a certain degree even in the presence of a disrupted regeneration epidermis. Fgf ligand presence and Lamb1a expression wane after completion of regeneration [48].

ECM components have been implicated in tissue regeneration in several recent studies, including tadpole tail bud regeneration, zebrafish heart regeneration, and newt heart regeneration [50–52]. It is generally challenging to dissect their biological functions during regeneration, as Laminins and other ECM molecules are often required during embryogenesis. For example, mice with null mutations in *Lama1*, *Lamb1*, or *Lamc1*, and zebrafish with mutations in *lamb1a*, *lama5*, or several other ECM components [41, 42, 53–55], each die at embryonic stages. However, as we reveal here, the regulation of Laminin is also critical in the context of regenerating large regions of bone, in a way we expect is both structurally to create an epidermal scaffold and chemically as a source of signaling factors. While our data implicate *fgf20a* as an inducer of *lamb1a* during fin regeneration, there may exist other regulatory inputs for this and distinct ECM factors. For example, Nagendran and colleagues recently reported in a morpholino-based study that *lama5* expression is under control of canonical Wnt signaling in the embryonic zebrafish fin epithelium [56]. Thus, it is possible that the regeneration epidermis is shaped by a network of growth factor-ECM regulatory interactions.

Interestingly, the estimated timing of teleost genome duplication precedes the dramatic rise of biological diversification in teleosts, according to paleontological evidence [38]. This is consistent with the hypothesis that gene duplication offers surplus genetic materials as origins of new biological functions. Recently, Rohner and colleagues reported that either of the two *fibroblast growth factor receptor 1* paralogs (*fgfr1a* and *fgfr1b*) is sufficient for embryogenesis in zebrafish, whereas loss-of-function in both paralogs is lethal [57]. Only one of the paralogs, *fgfr1a*, is specifically expressed in the skin of 30 day-old juveniles, where its function is required for normal adult scale development in adult zebrafish and carp. Here, we found that *lamb1a*, but not *lamb1b*, is induced during fin regeneration, where it is essential. We speculate that maintenance of two *lamb1* paralogs preserved viability in the setting of new mutations, permitting selection events that were favorable to adult regenerative potential. Comparison of the regulatory sequences of the *lamb1* paralogs, and other sets of paralogs with similar divergent expression upon injury, could help decode genetic modifications that have preserved or dampened regenerative capacity in the evolution of vertebrate species.

## Materials and Methods

### Animals

Adult zebrafish 3–5 months of age were used for most experiments. Animal density was maintained at 3–4 per liter in all experiments. *gup* ^*m189*^ and *s804* mutants were used to test complementation [41, 42]. *fgf20a* mutants (*dob*), *Tg(hsp70*:*dn-fgfr1)* ^*pd1*^, and *Tg(osterix*:*mCherry-NTRo)* ^pd46^ fish were described previously [20, 35, 48]. *sde1* mutants are referred to as *lamb1a* ^*pd110*^. For NVP-AEW541and NBI-31772 treatments, 16 wild-type animals (each) with 3 dpa fin regenerates were treated for 24 hours at 25°C in fish water containing either 2 μM NVP-AEW541 (Cayman Chemical) or 10 μM NBI-31772 (Calbiochem, 479830-5MG). Stocks were prepared in DMSO (5 mM and 20 mM) and control animals were treated with 0.04% DMSO. For FK506 treatments, after amputation, 12–15 fish were maintained at 33°C in 1L fish water containing 0.1 μg/ml FK506 (Sigma, F4679-5MG). Animals were fed every other day followed by a water change with fresh drug. FK506 was dissolved in DMSO for a stock solution of 5 mg/ml and control animals were treated with 0.004% DMSO. For SAG treatment, 8–9 animals were treated with either DMSO (0.05%) or SAG (5 μM; Cayman Chemical, CAS 912545-86-9) for two hours at room temperature every other day as described [31]. For juvenile experiments, 4 wpf *sde1* and *sde1/+* animals were transferred from 25°C to 33°C for a two-week treatment. All animal procedures were performed in accordance with Duke University guidelines, under protocol #A100-12-04.

### Mutagenesis and screen for fin regeneration mutants

EK or WIK strain males were mutagenized with ENU (3 mM) using published protocols (i.e. 1 hr/treatment/week for 4 weeks) [58] and mated to females of the WIK or EK strains to generate F1 families. A total of 108 F2 families and 423 F3 families were generated for identifying temperature-sensitive (TS) fin regeneration mutants. After fin amputation, F3 adult fish at 2–3 months of age were screened individually at 7 dpa at the restrictive temperature (33°C) for regeneration defects, using a dissecting microscope. Putative mutant families were out-crossed again to either EK or WIK strains to generate F4 and F5 families. A total of 9 mutants were subsequently selected from 25 putative F5 families for heritability, robustness of the defects, and survival rate at the restrictive temperature. For genetic mapping, homozygous mutant males of the F5 generation were crossed to F4 heterozygous females to generate pools of homozygous mutants and heterozygous siblings.

### Exome sequencing and genetic mapping

Genomic DNA was isolated from pools of 64 homozygous mutants and 51 heterozygous siblings using the Puregene Core Kit from Qiagen. For library preparation, 3 μg genomic DNA was sheared to 150–250 bp fragments using a Covaris sonicator. The sheared DNA was assessed on an Agilent chip to verify the size range. Library construction and exome capture were performed as described [36], using an Agilent early access SureSelect XT Zebrafish exome kit. Sequencing was performed using an Illumina HiSeq2000 with 100 bp paired-end (PE) runs. For the mutant pool, 47,116,257 paired-end reads were collected. For the het sibling pool, 44,813,293 paired-end reads were collected.

Fastq files for each mutant and heterozygous pool were concatenated and compressed before being uploaded to SNPTrack (http://genetics.bwh.harvard.edu/snptrack/). The appropriate pool sizes (64 vs. 51) were entered before starting analysis. We used 4 data sources to generate our “SNP universe”. The first is the Ensembl Release 71 VCF file containing 1,352,592 SNPs. The second data source is the Megason "Universe" containing 16,075,952 SNPs in a VCF file. The third data source is 6 whole genomes of various Zebrafish strains (AB, TLF, TUB, TUG, WKB, WKG) sequenced by the Harris lab. The final source of SNPs is sequencing data generated in the Poss lab by exome sequencing of 12 zebrafish pools and one whole-genome sequencing pool.

Preliminary analysis by SNPTrack revealed a 6.2 Mb region on chromosome 25 with a high homozygosity score. We identified 67,365 SNPs in the region. After filtering against our SNP universe, only 2 novel non-synonymous SNPs were found at the locations 32605161 and 34060320. Within this region, the percent usable on-target bases was 42%, and the mean target coverage was 51X for the mutant pool, while the percent usable on-target bases was 41%, and the mean target coverage was 47X for the het sibling pool.

For genetic mapping, we designed primers using Primer 3 (http://bioinfo.ut.ee/primer3-0.4.0/) to genotype individual SNPs. A high Resolution Melting (HRM)-based assay was used for genotyping. The assay was performed using the Roche LightCycler 480 and LightCycler 480 High Resolution Melting Master (Cat. No: 04909631001), follow the manufacturer's instructions. The primer sequences for SNP 32559436, SNP 32605161, and SNP 32681121 were listed in the S1 Table. All progeny from *sde1* x *sde1/+* mapping crosses were raised to 2–3 months old at 25°C, before phenotyping for regeneration defects at 33°C at 7 dpa.

### Histological assays

Whole-mount ISH with caudal fins was performed as described previously [22]. To generate digoxigenin-labeled probes for this study, we used *lef1* and *shha* cDNA fragments [30], and a partial 1 kb *lamb1a* cDNA fragment as templates (see S1 Table). Immunohistochemistry on sectioned fins was performed as described [30], using antibodies against aPKC (Santa Cruz Biotechnology, C-20-sc-216, 1:200), P63 (Santa Cruz Biotechnology, 4A4, 1:200), Zns-5 (ZIRC, 1:200), phosphorylated-Igf1r (Santa Cruz Biotechnology, sc-101703, 1:100), and Laminin (Sigma, L9393, 1:200). For EdU incorporation assays, EdU solution (10 mM) was injected intraperitoneally 60 minutes prior to collection of fin regenerates, which were then fixed and processed as described [59]. Frozen blocks were sectioned at 16 μm, mounted using Vectashield with DAPI, and examined by laser confocal microscopy (Zeiss 700).

For whole-mount Alizarin red staining, 4% PFA-fixed fins were rehydrated in 50% ethanol for 30 minutes. Fin tissues were then incubated in a solution with 0.5% KOH and 0.01% alizarin red S (Sigma A5533) for overnight staining. Next, fin tissues were bleached for 20 minutes at room temperature in a freshly made solution containing 1.5% H_2_O_2_ and 1% KOH. After three washes with water, tissues were transferred to 80% glycerol for storage and imaging.

### RT-qPCR

For each sample, fin tissues were collected from 4 individuals and homogenized in 1 ml Trizol (Invitrogen). cDNA was synthesized from 1 μg of RNA using Transcriptor First Strand cDNA Synthesis Kit (Cat. No: 04379012001). qPCR was performed using the Roche LightCycler 480 and LightCycler 480 Probes Master (Cat. No: 04887301001). Primer sequences and probes are listed in S1 Table. All samples were analyzed in biological and technical triplicate. Analysis was performed using the *ΔΔCT* method [60] against the level of *ribosomal protein L13a* (*rpl13a*) cDNA, which was found to be constant during fin regeneration.

## Supporting Information

S1 Fig Osteoblast patterning in *sde1* fin regenerates at 7 dpa. **(A)** *sde1* regenerates (right) are severely shortened at 7 dpa, comparable to a 3 dpa clutchmate sample (left). Osteoblasts (green) line the bone in proximal areas but accumulate in masses in distal regions. Scale bars, 50 μm. Red arrows indicate plane of amputation. (B) Whole-mount images of *sde1/+* and *sde1* regenerates at 7, 14, 21, and 28 dpa. Images from the same animal are shown here across different time points. Red arrows and dashed lines indicate plane of amputation. Scale bars, 1 mm. (C) Measurement of fin regenerates at different time points (n = 14 vs. 11; Student’s *t* -test, ***P < 0.001).
(TIF)

S2 Fig Long-term survival tests with regeneration mutants. Adult homozygous mutants (mt) and their heterozygous siblings (het) were incubated at the restrictive temperature (33°C) for two months. Animals were examined on a daily basis. *sde1* (*chc1*) mutation has no apparent impact on adult animals over a two-month period (n = 18 each).
(TIF)

S3 Fig *lamb1a* induction defines signaling competence in basal cells of the regeneration epidermis. (A) *shha* RNA expression is reduced in *sde1* regenerates at 4 dpa after a temperature shift from 25°C to 33°C at 3 dpa. (B) Similar results were observed in *sde1* regenerates at 5 dpa after a temperature shift at 4 dpa. Scale bars, 100 μm. Representative images from different samples are shown here.
(TIF)

S4 Fig Eight hours of 33°C treatment is sufficient to alter aPKC localization and reduce *shha* expression in *sde1* regenerates. (A) Antibody co-staining for aPKC (red) and P63 (green) in longitudinal sections of *sde1/+* and *sde1* fin regenerates after 8 hours of 33°C treatment at 3 dpa, indicating rapid loss of basal cell polarity. Scale bars, 50 μm. (B) *shha* RNA expression is also reduced in *sde1* regenerates after 8 hours of 33°C treatment (n = 5 each). Scale bars, 100 μm.
(TIF)

S5 Fig EdU incorporation assays in *sde1* and control regenerates. (A) Longitudinal sections of 5 dpa fin regenerates collected after 60 minutes of EdU incorporation. (B) Counting of EdU^+^ mesenchymal cells in distal fin ray area. Mesenchymal cell proliferation was grossly similar between *sde1/+* and *sde1* regenerates at 5 dpa after a temperature shift from 25°C to 33°C at 4 dpa. Scale bars, 50 μm. (n = 4, counts from three sections were averaged for each sample; Student’s *t* -test, NS, non-significant).
(TIF)

S6 Fig Tests of association between *fgf20a* and *lamba1*. (A) RT-qPCR analysis for levels of *fgf20a* in *sde1* fin regenerates. qPCR results were normalized to *rpl13a* and to the basal expression of *fgf20a* at 0 hpa. (n = 3; mean ± SEM; Student’s *t* -test, NS, non-significant). (B) Antibody co-staining for Laminin (red) and P63 (green) in longitudinal sections of wild-type and *dob* fin regenerates at 2 dpa. Scale bars, 50 μm. White arrows indicate where (low) levels of Laminin are detectable. Red arrows indicate plane of amputation. (C) RT-qPCR analysis *lamb1a* levels in 4 dpa wild-type fin regenerates after 24 hours of treatment with either Igf receptor antagonist NVP-AEW541 (2 μM), or Igf signaling agonist NBI-31772 (10 μM). (D) RT-qPCR analysis of *lamb1a* levels in 2 dpa *sde1/+* and *sde1* fin regenerates at the restrictive temperature. qPCR results were normalized to *rpl13a* and to the basal expression of *lamb1a* at 0 hpa. (n = 4; mean ± SEM; Student’s *t* -test, NS, non-significant).
(TIF)

S7 Fig FK506 treatment, but not SAG treatment, significantly increases the length of *sde1* regenerates. (A) Whole-mount images of *sde1/+* and *sde1* fin regenerates at 7 dpa after treatment of DMSO (0.04%) or FK506 (0.1 μg/ml). Scale bars, 1 mm. (B) Measurement of the fin length at 7 dpa (Student’s *t* -test, ***P < 0.001). (C) Fold change of fin regenerates at 7 dpa. Relative values are normalized to the length of *sde1/+* regenerates after DMSO treatment (mean ± SEM). (D) Whole-mount images of *sde1/+* and *sde1* fin regenerates at 6 dpa after treatment of DMSO (0.05%) or SAG (5 μM). (E) Measurement of the fin lengths at 6 dpa (Student’s *t* -test, NS, non-significant).
(TIF)

S8. Fig Impact of FK506 treatment on *shha* RNA expression in *sde1* regenerates. *shha* RNA expression was grossly similar between DMSO- and FK506-treated *sde1* regenerates at 7 dpa. Consistent with S3 Fig, *sde1* regenerates have a reduced level of *shha* expression. Scale bars, 100 μm. Representative images from different samples are shown here.
(TIF)

S1 Table Primer sequences used in this study. (DOCX)
