# Supplementary figures and images for: Transient laminin beta 1a Induction Defines the Wound Epidermis during Zebrafish Fin Regeneration
Source: PLoS Genet. 2015 Aug 25;11(8):e1005437. doi: 10.1371/journal.pgen.1005437 (PMC4549328; doi:10.1371/journal.pgen.1005437)

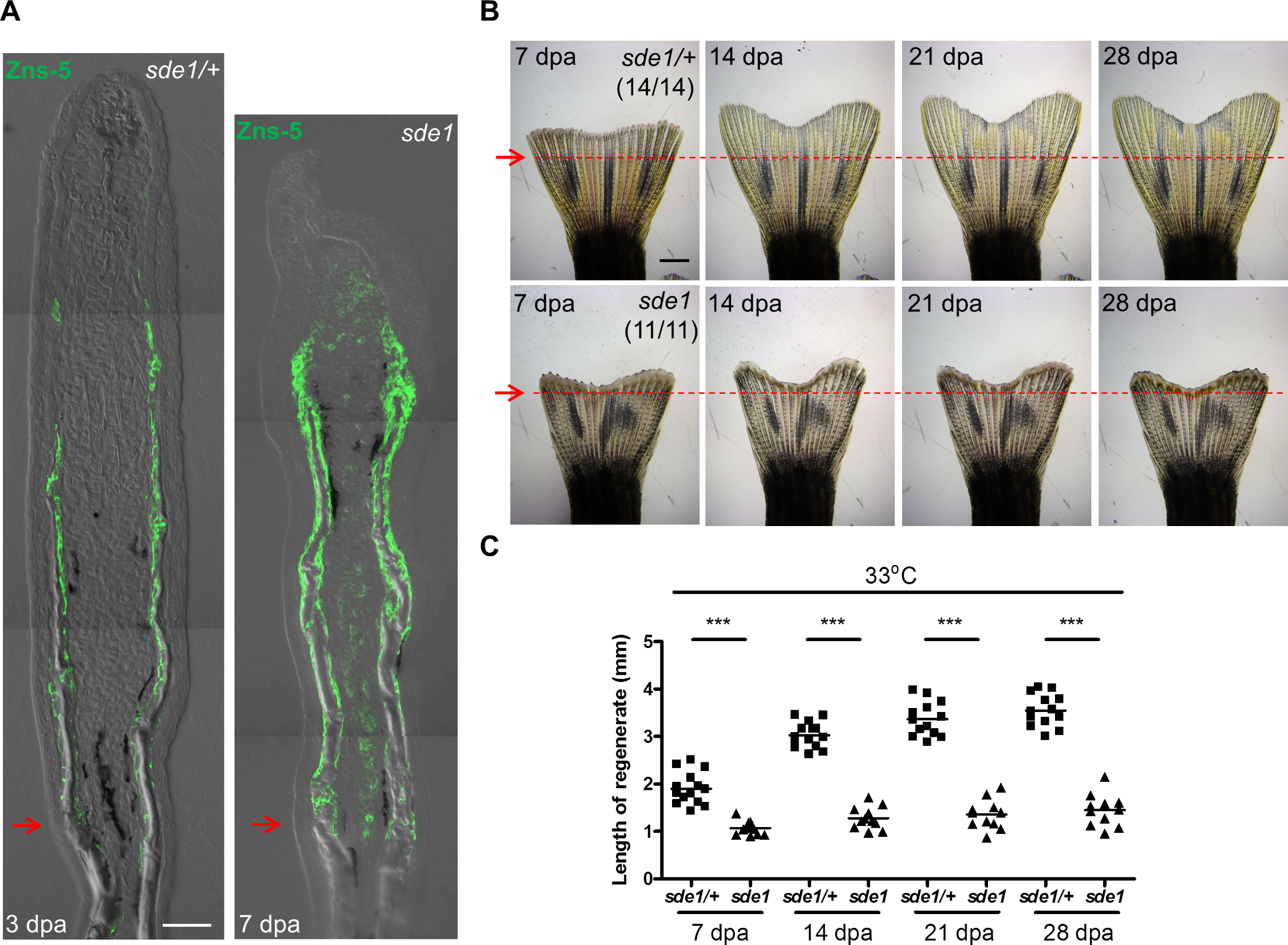

Supplement: S1 Fig — (A) sde1 regenerates (right) are severely shortened at 7 dpa, comparable to a 3 dpa clutchmate sample (left). Osteoblasts (green) line the bone in proximal areas but accumulate in masses in distal regions. Scale bars, 50 μm. Red arrows indicate plane of amputation. (B) Whole-mount images of sde1/+ and sde1 regenerates at 7, 14, 21, and 28 dpa. Images from the same animal are shown here across different time points. Red arrows and dashed lines indicate plane of amputation. Scale bars, 1 mm. (C) Measurement of fin regenerates at different time points (n = 14 vs. 11; Student’s t -test, ***P < 0.001). (TIF) [file pgen.1005437.s001.tif]

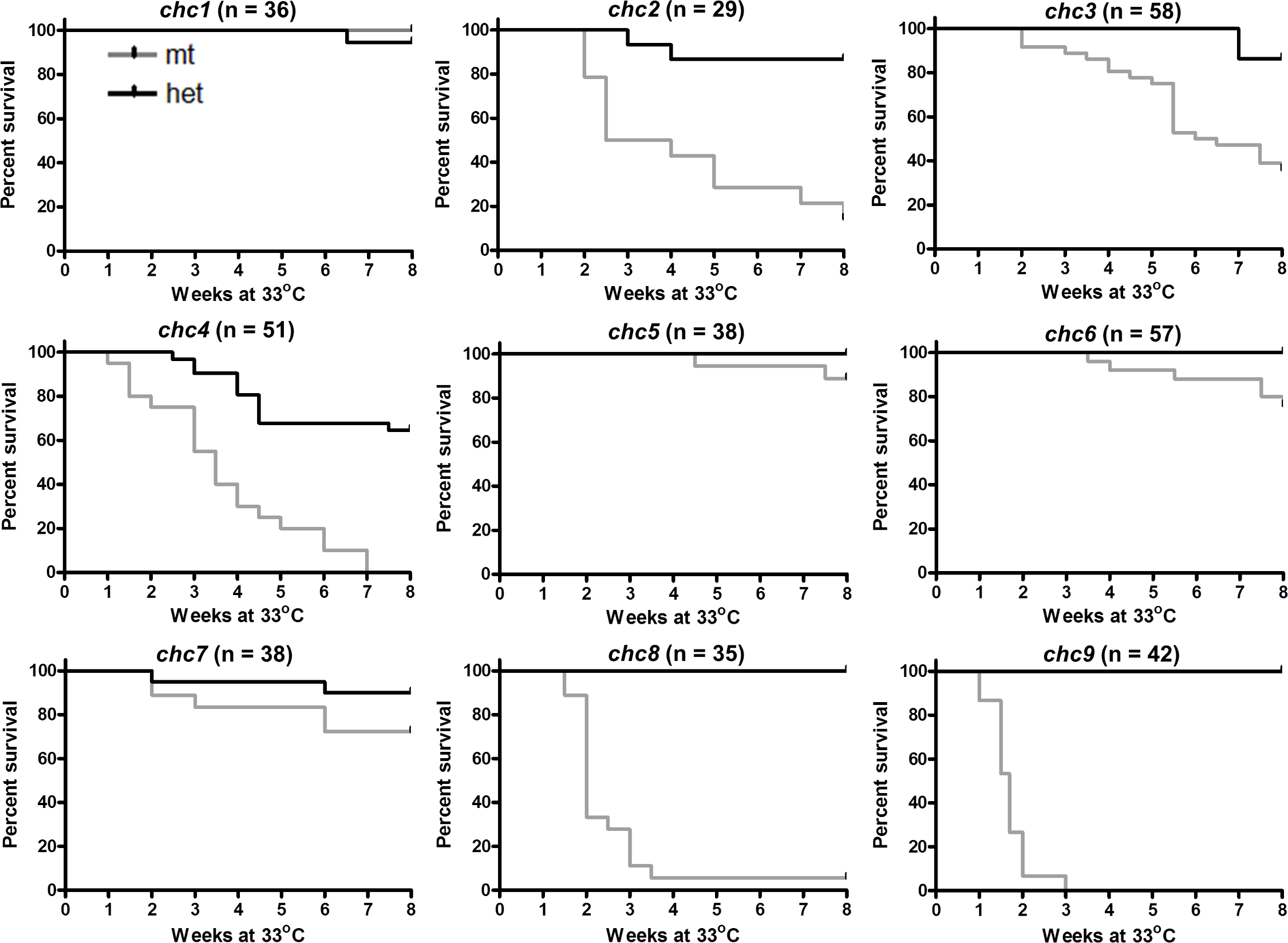

Supplement: S2 Fig — Adult homozygous mutants (mt) and their heterozygous siblings (het) were incubated at the restrictive temperature (33°C) for two months. Animals were examined on a daily basis. sde1 (chc1) mutation has no apparent impact on adult animals over a two-month period (n = 18 each). (TIF) [file pgen.1005437.s002.tif]

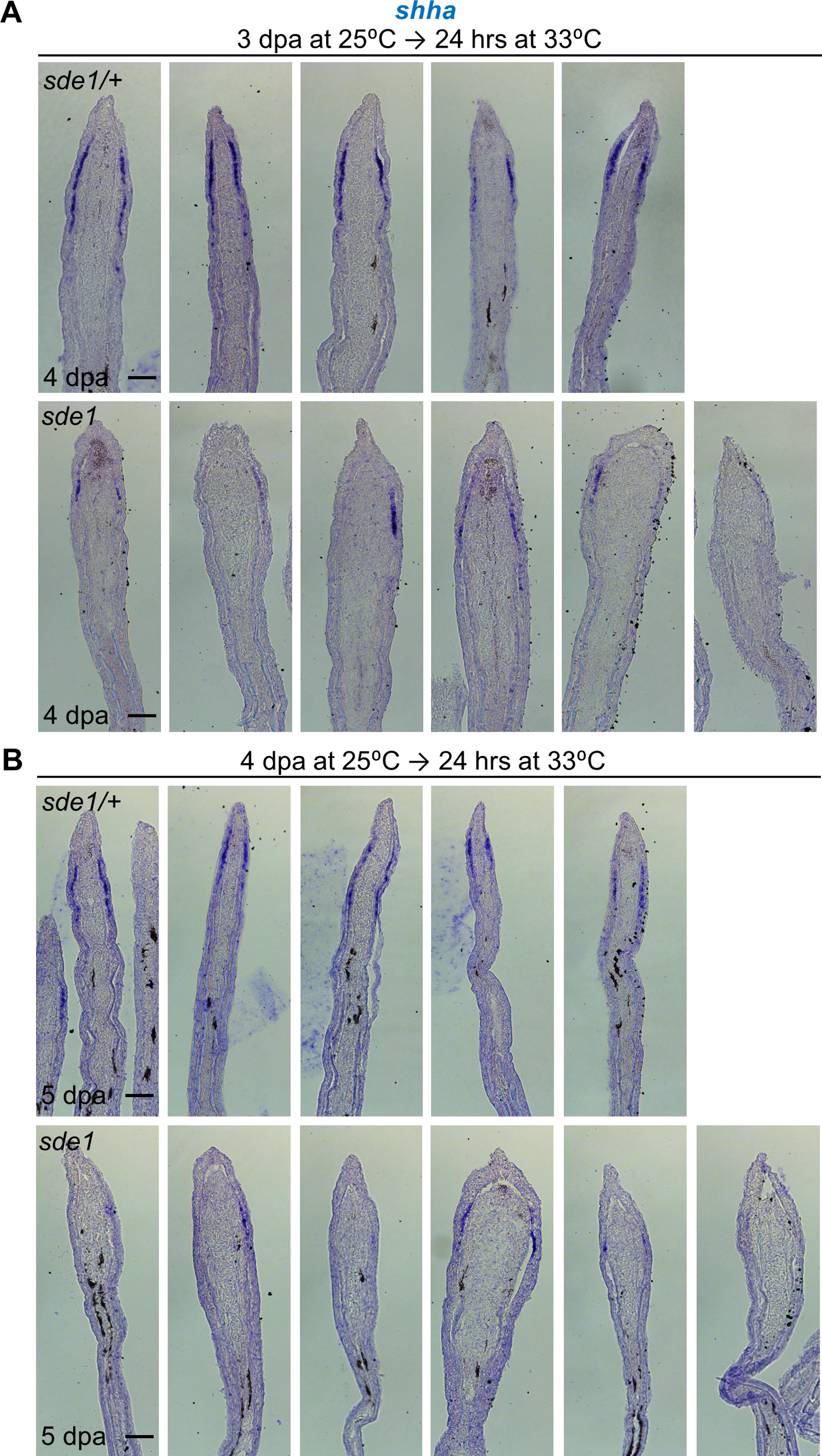

Supplement: S3 Fig — (A) shha RNA expression is reduced in sde1 regenerates at 4 dpa after a temperature shift from 25°C to 33°C at 3 dpa. (B) Similar results were observed in sde1 regenerates at 5 dpa after a temperature shift at 4 dpa. Scale bars, 100 μm. Representative images from different samples are shown here. (TIF) [file pgen.1005437.s003.tif]

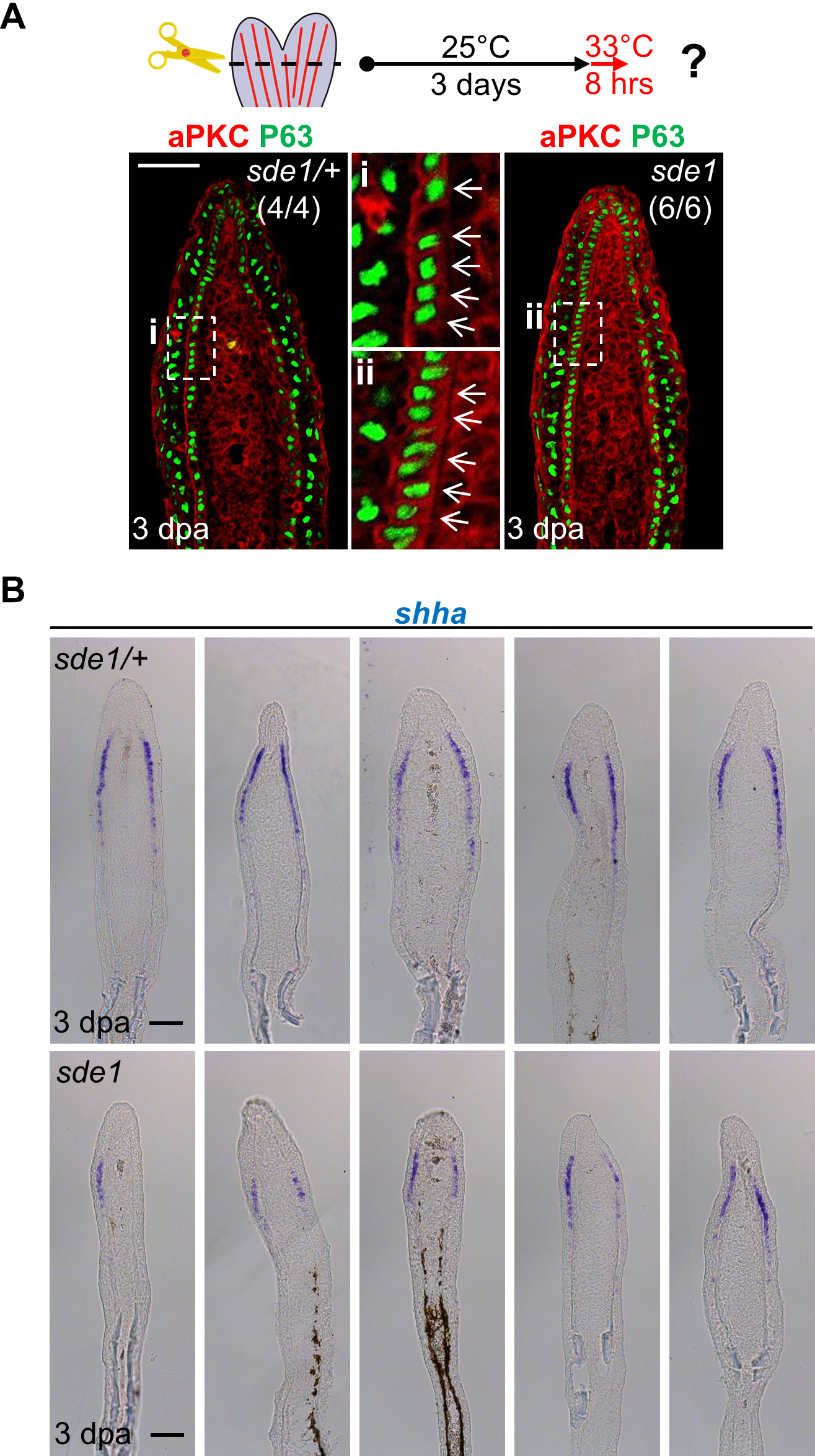

Supplement: S4 Fig — (A) Antibody co-staining for aPKC (red) and P63 (green) in longitudinal sections of sde1/+ and sde1 fin regenerates after 8 hours of 33°C treatment at 3 dpa, indicating rapid loss of basal cell polarity. Scale bars, 50 μm. (B) shha RNA expression is also reduced in sde1 regenerates after 8 hours of 33°C treatment (n = 5 each). Scale bars, 100 μm. (TIF) [file pgen.1005437.s004.tif]

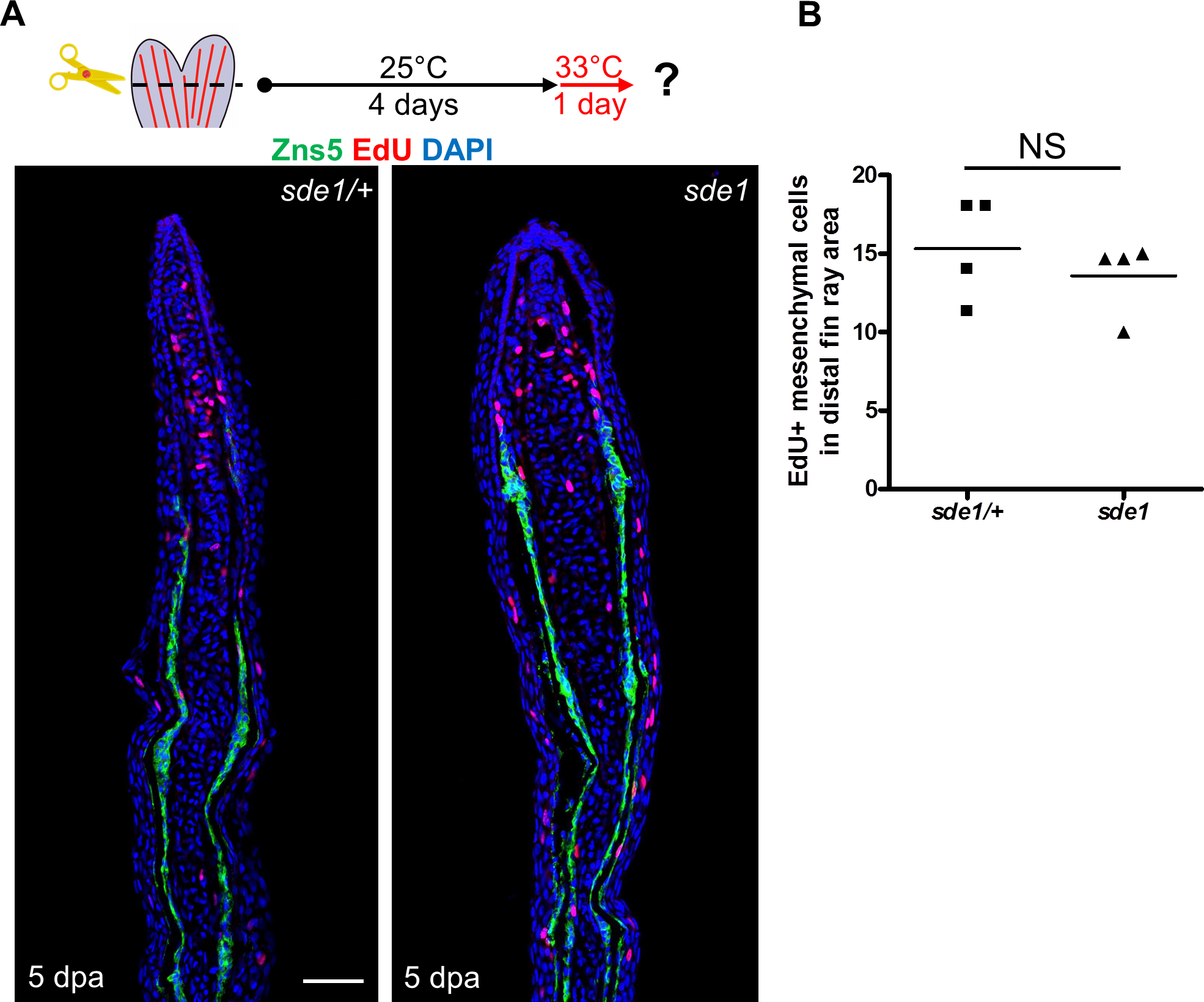

Supplement: S5 Fig — (A) Longitudinal sections of 5 dpa fin regenerates collected after 60 minutes of EdU incorporation. (B) Counting of EdU+ mesenchymal cells in distal fin ray area. Mesenchymal cell proliferation was grossly similar between sde1/+ and sde1 regenerates at 5 dpa after a temperature shift from 25°C to 33°C at 4 dpa. Scale bars, 50 μm. (n = 4, counts from three sections were averaged for each sample; Student’s t -test, NS, non-significant). (TIF) [file pgen.1005437.s005.tif]

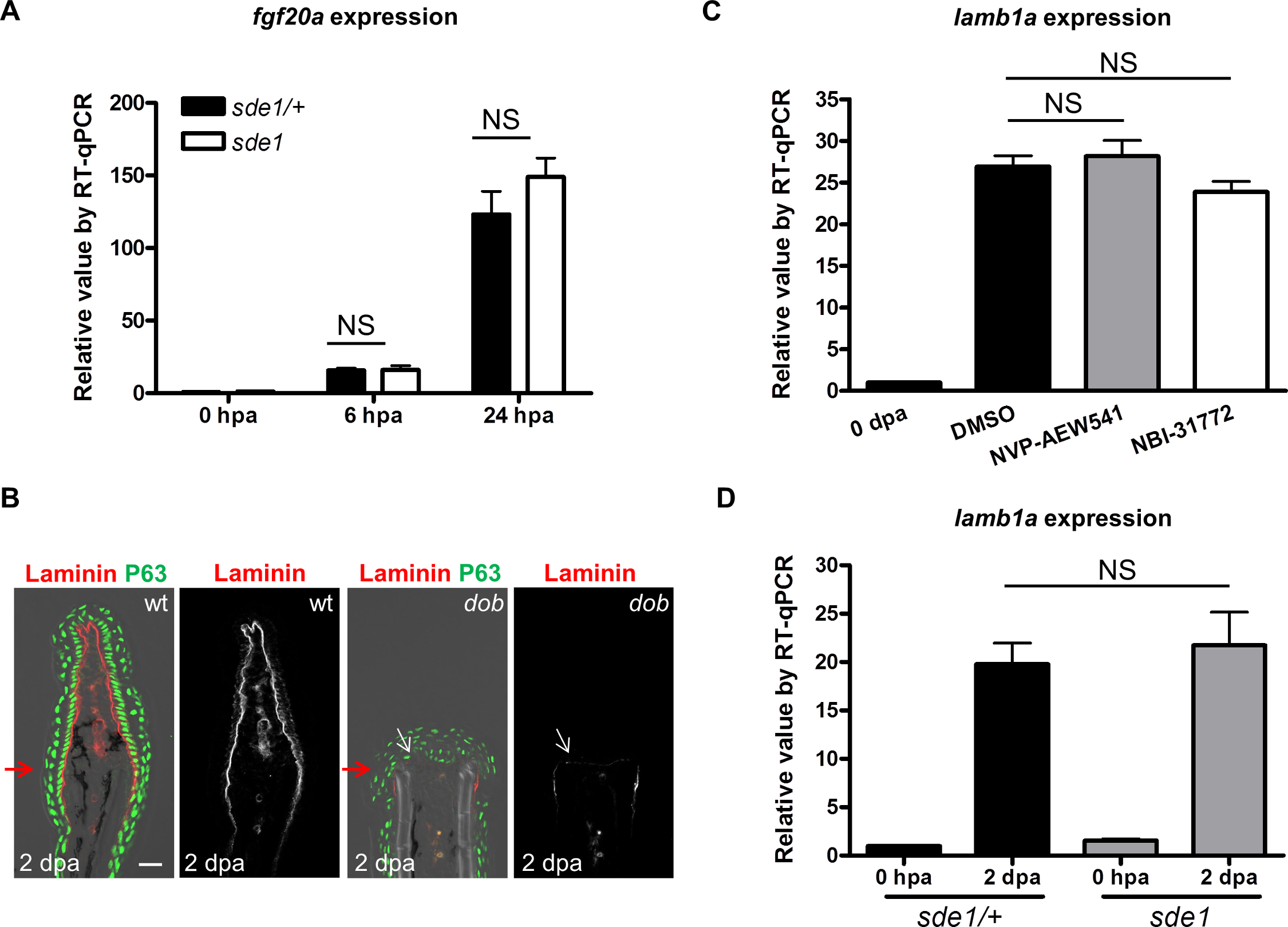

Supplement: S6 Fig — (A) RT-qPCR analysis for levels of fgf20a in sde1 fin regenerates. qPCR results were normalized to rpl13a and to the basal expression of fgf20a at 0 hpa. (n = 3; mean ± SEM; Student’s t -test, NS, non-significant). (B) Antibody co-staining for Laminin (red) and P63 (green) in longitudinal sections of wild-type and dob fin regenerates at 2 dpa. Scale bars, 50 μm. White arrows indicate where (low) levels of Laminin are detectable. Red arrows indicate plane of amputation. (C) RT-qPCR analysis lamb1a levels in 4 dpa wild-type fin regenerates after 24 hours of treatment with either Igf receptor antagonist NVP-AEW541 (2 μM), or Igf signaling agonist NBI-31772 (10 μM). (D) RT-qPCR analysis of lamb1a levels in 2 dpa sde1/+ and sde1 fin regenerates at the restrictive temperature. qPCR results were normalized to rpl13a and to the basal expression of lamb1a at 0 hpa. (n = 4; mean ± SEM; Student’s t -test, NS, non-significant). (TIF) [file pgen.1005437.s006.tif]

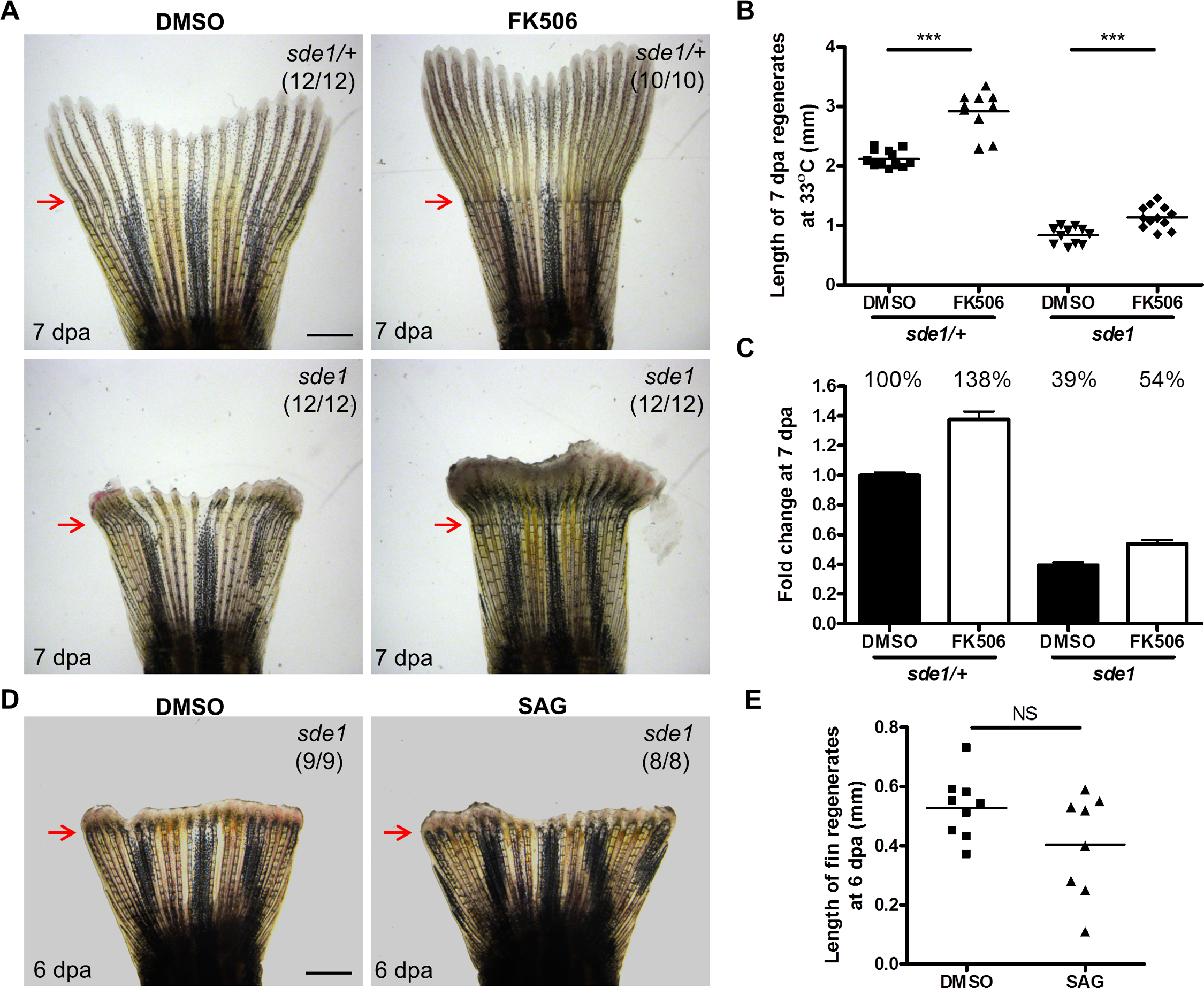

Supplement: S7 Fig — (A) Whole-mount images of sde1/+ and sde1 fin regenerates at 7 dpa after treatment of DMSO (0.04%) or FK506 (0.1 μg/ml). Scale bars, 1 mm. (B) Measurement of the fin length at 7 dpa (Student’s t -test, ***P < 0.001). (C) Fold change of fin regenerates at 7 dpa. Relative values are normalized to the length of sde1/+ regenerates after DMSO treatment (mean ± SEM). (D) Whole-mount images of sde1/+ and sde1 fin regenerates at 6 dpa after treatment of DMSO (0.05%) or SAG (5 μM). (E) Measurement of the fin lengths at 6 dpa (Student’s t -test, NS, non-significant). (TIF) [file pgen.1005437.s007.tif]

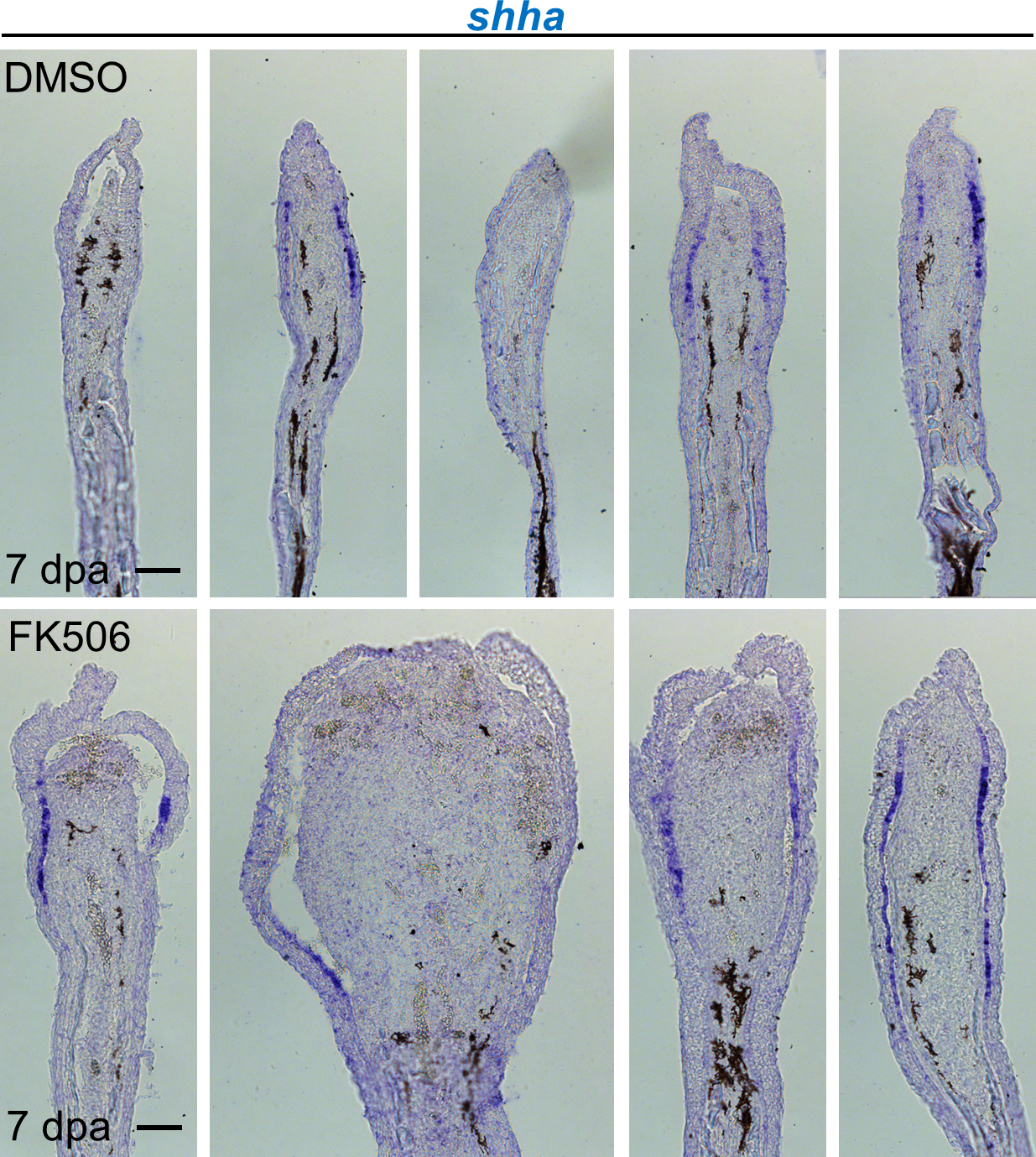

Supplement: S8. Fig — shha RNA expression was grossly similar between DMSO- and FK506-treated sde1 regenerates at 7 dpa. Consistent with S3 Fig, sde1 regenerates have a reduced level of shha expression. Scale bars, 100 μm. Representative images from different samples are shown here. (TIF) [file pgen.1005437.s008.tif]
