# Supplementary material for: Transient laminin beta 1a Induction Defines the Wound Epidermis during Zebrafish Fin Regeneration
Source: PLoS Genet. 2015 Aug 25;11(8):e1005437. doi: 10.1371/journal.pgen.1005437 (PMC4549328; doi:10.1371/journal.pgen.1005437)
Supplement: S1 Table — (DOCX) [file pgen.1005437.s009.docx]

S1 Table. Primer sequences used in this study

| qPCR (Universal ProbeLibrary System, Roche) | | | |
| --- | --- | --- | --- |
| Gene | Direction | Primer sequences | Probe No. |
| *lamb1a* | Forward | CCC CTG TCG ATG GAA CTG | 86 |
|  | Reverse | TAC ACA TAC AAT GAC CAT GAA CCA |  |
| *lamb1b* | Forward | CTC CCA ACC GCC TTA AAA C | 87 |
|  | Reverse | AAT CCA GCT GAA TGG TCA CA |  |
| *rpl13a* | Forward | GCA GAA TCT TCT GGA GGA CTG | 41 |
|  | Reverse | AAC CAT GCG CTT TCT CTT GT |  |
| *fgf20a* | Forward | TGG ATA GCG GAT TGT ATC TGG | 150 |
|  | Reverse | CAA ACT GCT CCC TGA ACA CA |  |
| HRM assay | | | |
| SNP 32559436 | Forward | ATT GAG CAC CCC TGC ATT AG | |
|  | Reverse | AGA GTC TTC TGG TGG CGT TG | |
| SNP  32605161 | Forward | TTT ATG GAC TCC GCA TGT TG | |
|  | Reverse | GAG CTG GGA GAT GTT TGC AC | |
| SNP  32681121 | Forward | GAG CTG ATT TAT GCT CGT CGT | |
|  | Reverse | TCC TAG CTG CTG CTT CTT CAG | |
| Cloning of partial *lamb1a* fragment (1170 bp) as a template for *in situ* probe | | | |
| *lamb1a* | Forward | GAA CCC TCA ACT GAG GGC GAG GTC | |
|  | Reverse | AAA ACC AAT GCC AGT CCA AG | |
